# Stability and Reactivity of Aromatic Radical Anions in Solution with Relevance to Birch Reduction

**DOI:** 10.1021/jacs.3c11655

**Published:** 2024-02-16

**Authors:** Tatiana Nemirovich, Brandon Young, Krystof Brezina, Philip E. Mason, Robert Seidel, Dominik Stemer, Bernd Winter, Pavel Jungwirth, Stephen E. Bradforth, H. Christian Schewe

**Affiliations:** † Institute of Organic Chemistry and Biochemistry of the Czech Academy of Sciences, Flemingovo nám. 2, 166 10 Prague 6, Czech Republic; ‡ Department of Chemistry, University of Southern California, Los Angeles, California 90089, United States; § Helmholtz-Zentrum Berlin für Materialien und Energie, Hahn-Meitner-Platz 1, 14109 Berlin, Germany; ∥ Fritz-Haber-Institut der Max-Planck-Gesellschaft, Faradayweg 4-6, 14195 Berlin, Germany; ⊥ J. Heyrovský Institute of Physical Chemistry, Czech Academy of Sciences, Dolejškova 3, 18223 Prague, Czech Republic

## Abstract

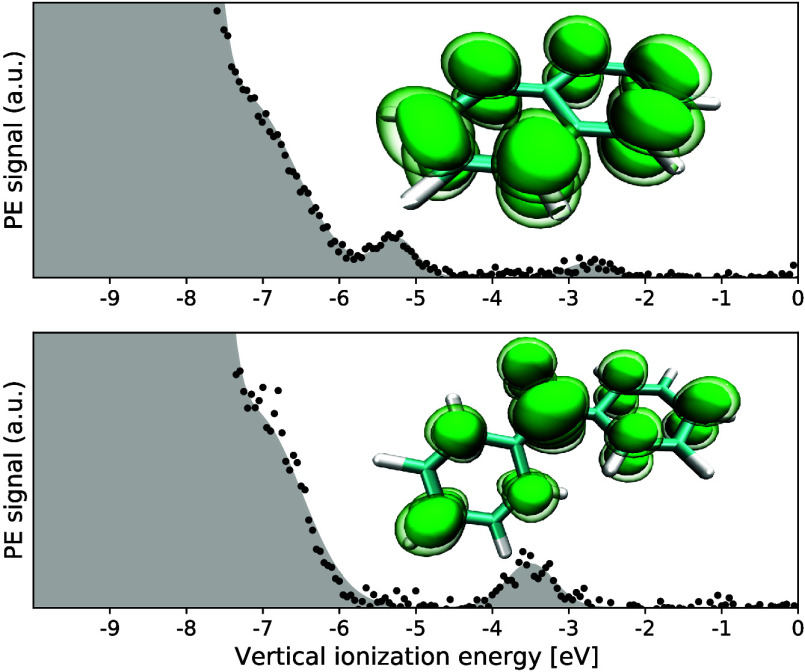

We investigate the electronic structure of aromatic radical anions in the solution phase employing a combination of liquid-jet (LJ) photoelectron (PE) spectroscopy measurements and electronic structure calculations. By using recently developed protocols, we accurately determine the vertical ionization energies of valence electrons of both the solvent and the solute molecules. In particular, we first characterize the pure solvent of tetrahydrofuran (THF) by LJ-PE measurements in conjunction with ab initio molecular dynamics simulations and G_0_W_0_ calculations. Next, we determine the electronic structure of neutral naphthalene (Np) and benzophenone (Bp) as well as their radical anion counterparts Np^–^ and Bp^–^ in THF. Wherever feasible, we performed orbital assignments of the measured PE features of the aromatic radical anions, with comparisons to UV–vis absorption spectra of the corresponding neutral molecules being instrumental in rationalizing the assignments. Analysis of the electronic structure differences between the neutral species and their anionic counterparts provides understanding of the primarily electrostatic stabilization of the radical anions in solution. Finally, we obtain a very good agreement of the reduction potentials extracted from the present LJ-PES measurements of Np^–^ and Bp^–^ in THF with previous electrochemical data from cyclic voltammetry measurements. In this context, we discuss how the choice of solvent holds significant implications for optimizing conditions for the Birch reduction process, wherein aromatic radical anions play crucial roles as reactive intermediates.

## 1 Introduction

Radical anions of aromatic hydrocarbons represent key reaction intermediates in areas spanning organic, inorganic, and organometallic chemistry.^1^ Among the well-known examples is the Birch reduction,^2^ wherein aromaticity in arenes is disrupted, leading to the formation of nonconjugated cyclohexadienes. These processes are exploited on synthetic and industrial scales to produce steroids, drugs, and medical compounds.^3^ Radical anions of aromatic hydrocarbons are typically formed in an electron transfer process from an alkali metal (usually assumed to take place via a solvated electron intermediate) into what has been assumed to be an unoccupied antibonding π* valence orbital of the aromatic hydrocarbons. In the Birch process, the creation of aromatic radical anion is considered the first crucial intermediate before a subsequent rate-determining protonation reaction takes place,^4^ facilitated by a suitable proton donor (typically an alcohol). While the classic Birch reduction is conducted in liquid ammonia, various approaches have been pursued recently to identify solution and solvent environments where Birch reduction may be performed at room temperature.^5,6^ The search for new solvents for the Birch reduction process is driven also by the fact that in contrast to liquid ammonia most organic solvents typically do not accommodate solvated electrons in large enough concentrations for extended periods of time. While numerous studies have explored the stability of aromatic radical anions in the gas phase,^7−10^ binding energies of the valence electrons of polycyclic aromatic radical anions in the solution phase are hitherto unexplored. Understanding the electronic structure of aromatic radical anion species may be instrumental in proposing new solvent environments for Birch-like reactions and for understanding the stereochemistry of the resulting product species.^11^

The naphthalene radical anion (Np^–^) and the benzophenone radical anion (Bp^–^) are two prototypical aromatic hydrocarbon anions that are of general interest for many chemical processes. First, they are used as reductive compounds in specialized synthesis, e.g., in the pharmaceutical industry^12^ since the anions form in up to molar concentrations in many different solvents starting with the corresponding neutral (naphthalene (Np) and benzophenone (Bp)). Second, the radical anions are stable for hours if the temperature-stable experimental setup is kept under oxygen- and water-free conditions. Beyond organic chemistry, both species are intriguing subjects to study solvation phenomena: while both Np^–^ and Bp^–^ are stable anions in solution, only Bp^–^ forms a stable radical anion in the gas phase,^7^ while Np^–^ occurs only as a metastable shape resonance.^13^ The reactivity of both radical anions has also been quantified by determining their reduction potentials using cyclic voltammetry (CV) in tetrahydrofuran (THF).^1^

We employ the liquid microjet (LJ) technique in conjunction with soft X-ray and VUV photoelectron spectroscopy (PES) to directly investigate the electronic structure of volatile solutions containing the above-mentioned anions.^14−16^ This method is well established for water and aqueous solutions, while extensions to cryogenic and nonpolar liquids such as liquid argon and methane or most recently liquid ammonia and benzene solutions have been accomplished.^17−20^ The peaks in the measured spectra correspond to the vertical ionization energies (VIEs) of both the solvent itself and the solute species directly probing the electronic structure of the initial equilibrium state of the studied systems.^21^ Thus, the LJ-PES technique is well suited to investigate the singly occupied molecular orbital (SOMO) of the radical anions which enables the determination of the stability in terms of vertical detachment energies (VDEs). From the onsets of the liquid’s highest occupied molecular orbital (HOMO) or solute anion’s SOMO spectral features, we can estimate the adiabatic ionization and detachment energies (AIE and ADEs), enabling the quantification of reduction potentials for neutral or anionic solutes and relating thus to their chemical reactivity. Moreover, LJ-PES is inherently sensitive to changes in the solute charge states,^22,23^ identified by electron binding energy (eBE) shifts or changes in the peak shape of the valence band (VB) and core-level spectral features.

In the present work, we report LJ-PES measurements and electronic structure calculations of Np and Bp as well as their radical anionic counterparts of Np^–^ and Bp^–^ in the solvent environment of THF. The electronic open-shell character of the anionic species gives rise to a number of challenges. First, the initial state and final state contributions to the eBEs lead to separated features in the PE spectra which require a detailed analysis and assignment of the measured PE spectral features. Second, the electronic structure calculations of the photoionization energies require multireference approaches to accurately grasp the complex nature of the excess electron being delocalized over the conjugated systems.^24^

The paper is structured as follows: We characterize pure liquid THF for the first time to quantitatively determine the electronic structure of a solvent that is widely used in organic synthesis. This is done by using LJ-PES in combination with ab initio molecular dynamics (AIMD) simulations followed by G_0_W_0_ calculations. Next, we establish the electronic structure of Np/Np^–^ and of Bp/Bp^–^ in THF. We determine accurately their VIEs employing recently developed protocols.^20,25,26^ Accompanying electronic structure calculations enable us to model the experimental PE spectra of these species in order to investigate solvent effects and to perform a proper orbital assignment of the measured PE features. Moreover, the delocalization pattern of the excess electron and the gas–liquid shift from the theoretical calculations provide us with insights into the mechanisms leading to the stabilization of these radical anions upon solvation. In this context, the Np/Np^–^ systems are discussed in relation to our previous computational results on benzene and its radical anion (Bz/Bz^–^).^27,28^ Finally, we compare the reduction potentials extracted from the present LJ-PES measurements with literature values from CV measurements,^1^ which allows us to relate directly to the chemical reactivity of these radical anions in solution.

## 2 Methods

### 2.1 Experiments

The experimental procedures consisted of three parts: solution preparation, conduction of LJ-PES measurements, and data analysis. We will only summarize the relevant parts, while a much more detailed description and characterization is provided in the experimental procedures Section (1) in the Supporting Information (SI).

THF solutions were prepared in a stepwise manner: first, THF was purified using a distillation unit that rigorously removes trace water with alkali metal using benzophenone as an indicator. Second, defined amounts of solid naphthalene (Sigma-Aldrich, 99%) or benzophenone (Sigma-Aldrich 99%) were dissolved in the purified THF. To generate the radical anion solutions potassium (Sigma-Aldrich, 98%) was added in excess underneath an argon atmosphere. The radical anion solutions were filtered in a nitrogen glovebox before they were transferred to the cryostat unit under airtight conditions for measurements.

**Figure 1:**
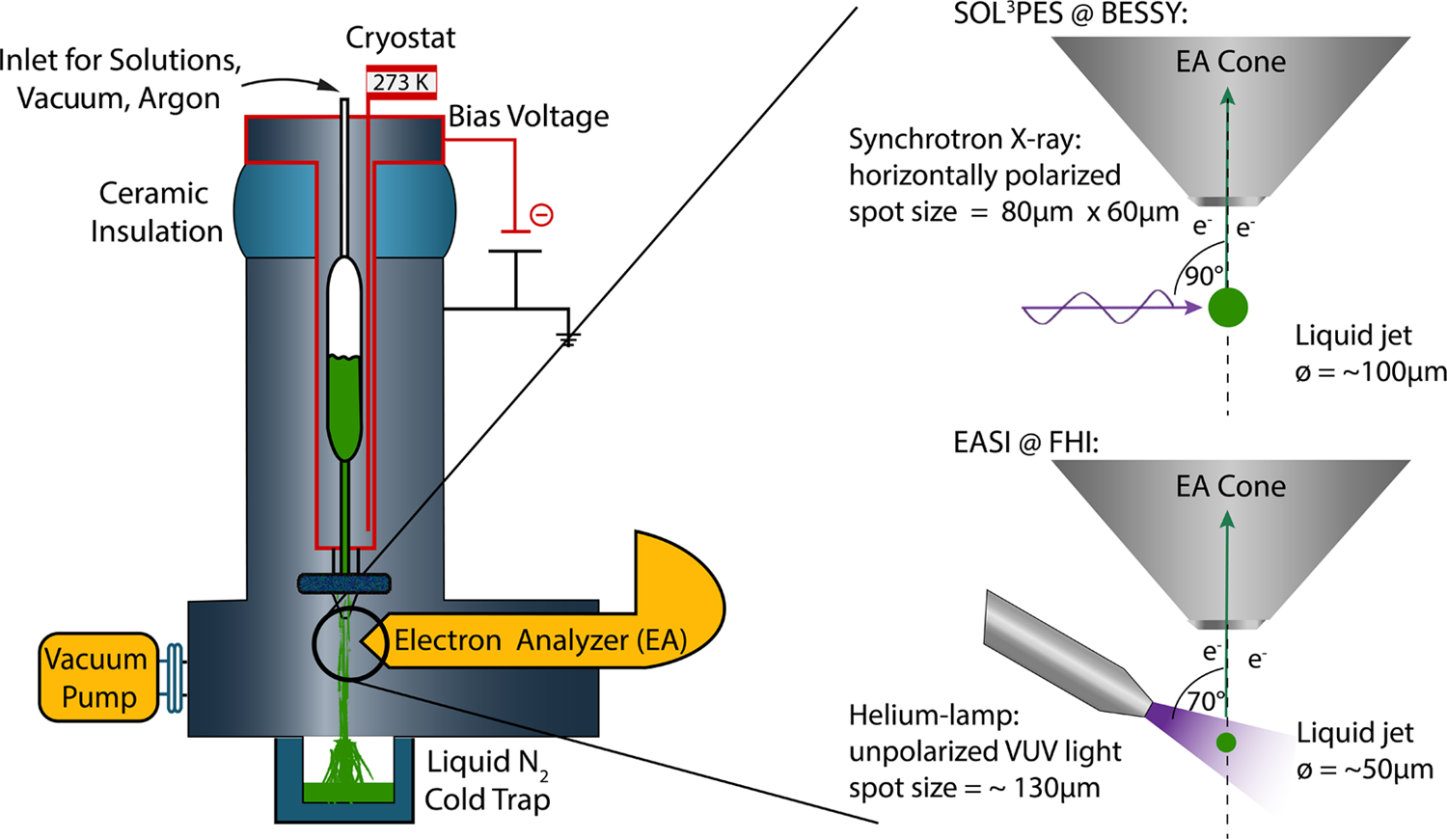
Sketch of the experimental setup: (left) cryostat unit being mounted from the top onto an insulating ceramic flange enabling the application of a bias voltage between the liquid jet and the hemispherical electron analyzer mounted from the right. A cold trap is mounted at the bottom effectively freezing out the liquid jet which has disintegrated into droplets. (Top right and bottom) Experimental geometries given at the two instruments: SOL^3^PES at BESSY II and EASI at FHI, respectively. Details are discussed in the text and the SI.

Figure 1 (left) depicts the cryostat unit which encases the sample cylinder in a cold ethanol bath which is cycled by a commercial chiller unit and kept at a constant temperature of ∼273 K. Liquid microjets were routinely generated by applying pressure of 2–3 bar of argon at the headspace of the sample cylinder which pressed the solutions into the vacuum chamber via a quartz capillary (inner diameter of 50 μm), which was affixed to the outlet of the filter. The cryostat unit is mounted on top of a ceramic flange, ensuring electric insulation of the cryostat unit and thus the liquid jet to the main instrument. We applied a bias voltage *U* between the liquid jet and the electron analyzer generating an electric field that accelerates photoelectrons toward the hemispherical electron analyzer.

We performed LJ-PES measurements at two different instruments, first at the U49/2-PGM-1 beamline^29^ at the BESSY II electron storage ring operated by the Helmholtz-Zentrum Berlin für Materialien and Energie using the SOL^3^PES setup.^30^Figure 1 (top right) depicts the assembly geometry shown from the top perspective: the LJ runs vertically downward, while the linearly polarized X-ray beam (polarization axis is in the horizontal plane) illuminates the LJ in 90°. Photoelectrons (PE) were measured using a Scienta Omicron HiPP2 hemispherical electron analyzer mounted in the horizontal plane 90° relative to the LJ and 90° to the X-ray beam.

Valence band PE spectra were first recorded at BESSY II with a photon energy of *h*ν = 123.464 ± 0.004 eV with an overall instrumental energy resolution of 30 meV (fwhm) determined by the convoluted uncertainty of the electron analyzer (25 meV) and the beamline (16 meV). The photon energy of the beamline was calibrated on a daily basis by measuring an electron-yield X-ray absorption spectrum generated by the integration of the electron emission arising from the 2p3p3s Auger decay channel of an argon resonance at *h*ν = 246.928 ± 0.004 eV. For calibration, we used the 10% contribution from the respective second harmonic undulator light (*h*ν = 246.928 eV) present in addition to the fundamental light *h*ν = 123.464 eV. The beamline exit slit defined the rectangular shape of the 40 × 60 μm^2^ (horizontal × vertical) X-ray spot size.

For reproducibility, measurements were later conducted using the EASI setup^31^ at the Fritz-Haber Institute (FHI) of the Max-Planck Society. Figure 1 (bottom right) depicts the geometry used at FHI: the liquid jet runs vertically downward 90° to the horizontally mounted helium plasma light source and the hemispherical electron analyzer (Scienta Omicron HiPP3) while the angle between the latter two components is 70°.

The VUV helium plasma lamp emitted unpolarized light which is supplied through a monochromator and a quartz capillary. The He–II α line provided a photon energy of *h*ν = 40.814 ± 0.002 eV, where the intrinsic width of the atomic line determines the uncertainty. The beam spot size was determined at the point of incidence with the liquid jet and had an approximate diameter of 300 μm. Using a pass energy of 20 eV, the hemispherical analyzer energy resolution was found to be about 40 meV.^25^

In order to separate the liquid phase from the gas-phase PE spectral contributions, a negative bias voltage *U* (−50 V for SOL^3^PES and −25 V for EASI) was applied. This enables us to perform an absolute binding energy calibration^25,32^ by recording the spectral features of the valence band as well as the low-energy tail (LET) curve. These protocols^25^ allow the determination of the eBEs (and correspondingly VIEs^33^ to be consistent across different measurements despite certain parameters—such as the photon energy or the effective bias voltage—might change).

### 2.2 Electronic Structure Calculations: Ab Initio Molecular Dynamics and G_0_W_0_ Calculations

A configurational sampling of the geometries of the THF molecule in the gas phase at a constant temperature of 300 K was obtained through a classical ab initio molecular dynamics simulation using the CP2K software, version 2022.1.^34^ The electronic structure was solved on the fly using the Quickstep^35,36^ DFT module of CP2K. We used the revPBE0-D3 hybrid density functional^37−40^ to represent the valence electrons and the Goedecker–Tetter–Hutter pseudopotentials^41^ to represent the 1s core electrons of carbon and oxygen atoms. The Kohn–Sham orbitals were expanded into the TZV2P-GTH basis^35^ set and, at the same time, a plane-wave basis with a 600 Ry cutoff was used to represent the electron density. The system was described in open boundary conditions, which was achieved by centering the molecule in a 12 Å wide cubic box and employing the Possion wavelet solver for electrostatic interactions, as implemented in CP2K. In addition, we employed the auxiliary density matrix method^42^ with a pFIT3 fitting basis set to accelerate our calculation. The atomic nuclei were propagated for a total simulation time of 10 ps using the Verlet propagator and an integration time step of 0.5 fs. Canonical conditions were imposed using a local stochastic velocity-rescaling thermostat^43^ with a time constant of 50 fs.

For the G_0_W_0_ calculation,^44^ which is used to access physically meaningful one-electron binding energies to model the electronic density of states, we selected a subset of ab initio molecular dynamics geometries regularly separated in time by a stride of 10 fs. The calculations of the G_0_W_0_ energy corrections were performed again using CP2K and started from Kohn–Sham orbitals obtained with identical electronic structure settings as the ab initio molecular dynamics simulation (see Section 2A in the SI for more details).

### 2.3 Modeling Photoelectron Spectra and Ionization Energies of Aromatic Radical Anions

The calculation of ionization energies of two radical anion species (naphthalene and benzophenone) as well as their neutral counterparts is described in this section. In this study, we calculated the lowest ionization energy (corresponding to ionization from the HOMO orbital for the neutral species and the SOMO orbital for the anionic species) as the energy difference between the ground states of the initial and ionized species. It is important to clarify that in the case of radical anions, “initial species” refers to the anionic state and “ionized species” to the neutral state. For ionization energies of neutral molecules, the “initial species” is the neutral state, and the “ionized species” refers to the cationic state postionization. For higher ionization energies, we calculated the energy difference between the ground state energy of the initial species and the excited states of the ionized species^45^ (see Section 4A in the SI for more details about theoretical methods).

The neutral and anionic structures were first preoptimized in a PCM of tetrahydrofuran using the B3LYP hybrid DFT functional^46^ equipped with the D3BJ dispersion correction^47,48^ employing the aug-cc-pVTZ^49^ basis set. These structures were then used for gas-phase single-point CASSCF calculations. The final gas-phase single-point energies for ground states of parent and ionized species, as well as for the first five excited states of the ionized species were obtained using the SC-NEVPT2 perturbation^50−52^ over CASSCF with the same basis set. The low-ionization-potential region of aromatic compounds typically includes only π-electron ionization bands.^53^ Therefore, the π molecular orbitals (MOs) were included in the complete active space for the CASSCF calculations, resulting in a CASSCF-(10-10) active space for Np and CASSCF-(11-10) for Np^–^. In the same manner, the active space for Bp included all of the 14 π MOs, resulting in CASSCF-(15-14) for Bp^–^ and CASSCF-(14-14) for Bp.

The solvation energy for each of the investigated aromatics was evaluated at the B3LYP/aug-cc-pVTZ^49^ level of theory with the D3BJ dispersion correction,^47,54^ employing the nonequilibrium PCM (NE-PCM) formalism as implemented in the Gaussian16^55^ program package. We calculated the solvation energy of the parent structure before ionization by taking the difference between the energy of the optimized parent species in the THF PCM model and the gas-phase energy of the same structure without any geometry relaxation. To obtain accurate values of the solvation energies, the calculations were performed separately for each of the studied excited states. It is important to note that since no geometry relaxation occurs during photoionization, we performed all calculations on the same preoptimized geometries of the initial species before ionization, employing PCM of THF. The obtained CAS-SCF gas-phase photoionization energies were then shifted by the corresponding gas–liquid shift obtained separately for each of the states.

## 3 Results

### 3.1 Characterization of the Solvent THF

In order to characterize the valence band electronic structure of the solvent THF we first investigate THF in the gas phase. This enables us to relate our findings to previous investigations by adopting, e.g., orbital assignments from the gas phase to the liquid phase. It also allows us to determine the gas-to-liquid peak shift which informs on the effect of solvation on the ionization process and whether there are any differential shifts between different molecular orbitals.

**Figure 2:**
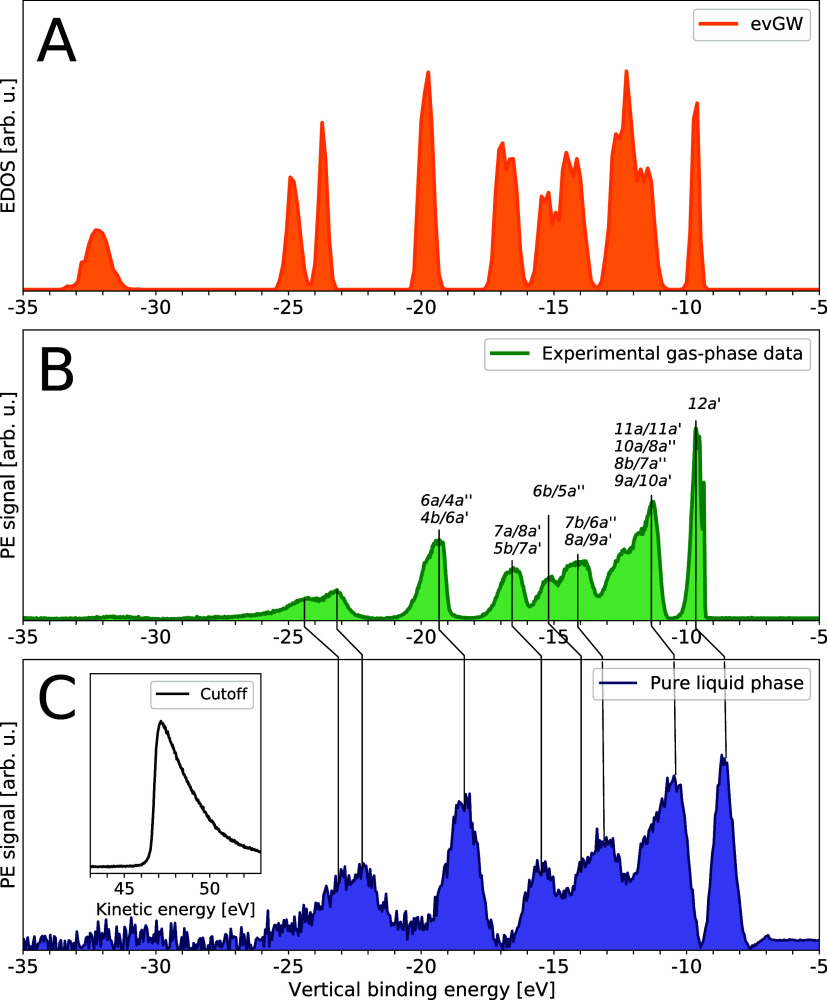
Valence band spectra of THF: (A) electronic density of states calculated using evGW; (B) gas-phase photoelectron spectrum of THF with assignments are from ref (56); (C) liquid-phase PE spectrum of THF with a salt concentration of 0.1 mol/L TBA^+^PF_6_^–^. By applying a bias voltage of *U* = −50 V, the low electron energy cutoff is measured (inset) together with all valence band spectral features of interest.

Figure 2B depicts the gas-phase PE spectrum of THF recorded using the SOL^3^PES instrument at BESSY II. For this, we moved the liquid jet out of the synchrotron light beam such that only THF molecules that had evaporated from the liquid surface were ionized. The binding energy axis has been calibrated using the gas–liquid phase spectrum shown in Section 2B in the SI. Following ref (56), we assigned the spectral features to MOs from which ionization takes place as a function of increasing eBE. The peak positions of the individual spectral features (stated in Table S1 in the SI) are in good agreement with prior experimental work which also considered ionization to be approximately 20 eV above the first IP.

The gas-phase THF experimental peak positions are closely reproduced by evGW calculations, as shown in Figure 2A. Intensities of the spectral features differ between experiment and theory as the calculation at this instance examines the EDOS without any reference to experimental cross sections; unlike the experimental spectrum, the calculated EDOS integrates to the number of states. The THF molecule is known to exhibit an out-of-plane ring puckering deformation which allows the existence of two unique potential energy minimum geometries that can be switched between each other through a pseudorotational motion. Giuliani et al.^57^ identified the two conformations of C_2_ (twisted) and C_*s*_ (envelope) symmetries and estimated their simultaneous populations at 298 K at a ratio of 0.55–0.45, respectively. Consistently, our AIMD trajectory samples frequent transitions between the corresponding potential energy wells, which implies low barriers for the process. As such, the calculated EDOS takes into account these deformations and the data shown in Figure 2A represents the average density of states over all possible, thermally available geometries. This is also the case with the experimental spectrum. In principle, one might expect that a deeper insight is revealed from calculations to obtain the individual contributions to the spectra originating from the individual potential wells surrounding the equilibrium conformers. However, we show in Section 2C in the SI that there is very little variation in the EDOS curves for different THF conformers within the available statistics.

Figure 2C depicts the liquid-phase spectrum (black line) from a pure liquid THF microjet with a salt concentration of 0.1 mol/L TBA^+^PF_6_^–^. When comparing the gas-phase valence band spectrum to the liquid phase data, we observe that all spectral features show practically the same binding energy shift of ∼1.3 eV compared to their gas-phase counterpart—a detailed and individual comparison is given in Table S1 in the SI. The shift is graphically displayed in Figure 2 by the displaced vertical black lines connecting each individual valence band peak from the gas-phase spectrum of panel B to its corresponding peak from the liquid-jet PE spectrum in panel C. In addition, we notice a small broadening of ∼0.1 eV due to the solvation of the already vibronically broadened spectral features.

By applying a bias voltage of *U* = −50 V we employ the protocols to determine absolute binding energies by measuring the low kinetic energy cutoff as well as all spectral features of interest.^20,25,32^ Thus, we determine the VIE of the liquid HOMO of THF to be VIE_liq_^THF^ = 8.45 ± 0.1 eV, while the averaged literature value for the gas-phase is VIE_gas_^THF^ = 9.71 ± 0.03 eV,^56,57^ resulting in a gas–liquid shift of ΔBE^THF^ = 1.28 eV. For comparison, we apply a Born–Haber cycle^20^ to provide an estimate for the gas–liquid shift ΔBE_BH_^THF^ for ionizing a neutral THF molecule in the solvent of THF. We employ the high-frequency dielectric constant ε_r_ given by the square of the refractive index ε_r_ = *n*^2^ = (1.401)^2 ^^58^ and an average molecule–molecule distance of 5.25 Å^59^ in the liquid phase. This results in a gas–liquid shift value of ΔBE_BH_^THF^ = 1.36 eV which agrees well taking into account that the THF structure deviates from a sphere.

### 3.2 Experimental Spectra of Np/Np^–^ and Bp/Bp^–^ in THF

In the following we evaluate and interpret the PE spectral features measured for the Np/Np^–^ and Bp/Bp^–^ species in THF adopting spectral assignments and concepts from gas-phase radical anion PES and UV–vis absorption spectroscopy of neutrals in solution wherever possible. Gas-phase Np has a VDE of 8.144 eV,^64^ while its electron affinity (EA) is negative when isolated in vacuum. On the other hand, the negative ion Np^–^ is known to be stabilized in the weakly polar solvent, THF, giving a strongly colored green solution.^1^ Bp has a higher ionization energy in the gas phase but supports a stable negative anion Bp^–^ in vacuum with a VDE between 0.7 and 1.0 eV.^65^ However, the binding energies of the valence electrons in these or any other simple polycyclic aromatic anion in the solution phase are hitherto unknown.

**Figure 3:**
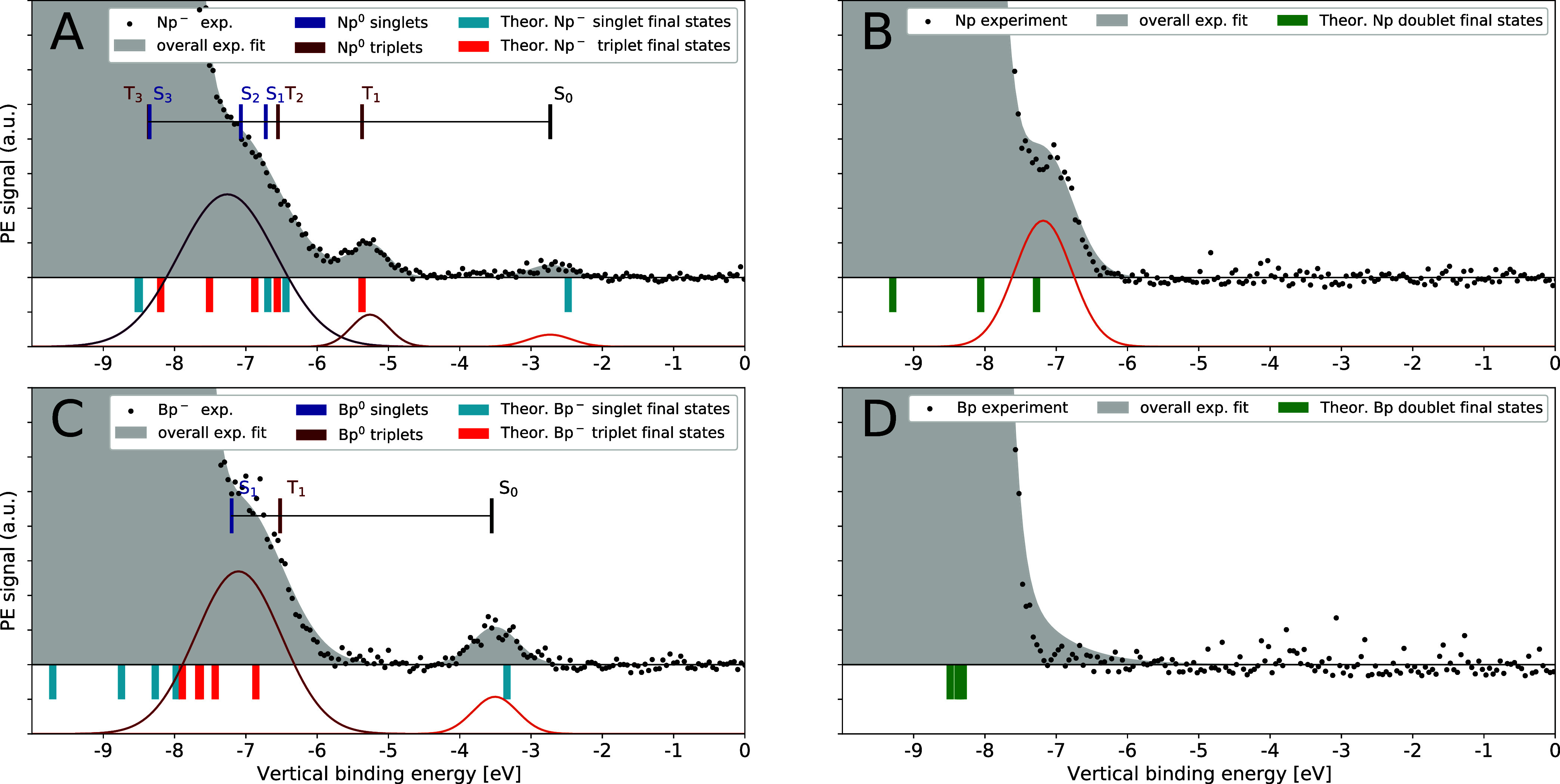
Experimentally measured PE spectra (dots) for neutral and radical anion aromatics dissolved in THF: (A, B) Valence band spectra of the naphthalene radical anion (Np^–^) and neutral naphthalene (Np) in THF; (C, D) spectra of the benzophenone radical anion (Bp^–^) and neutral benzophenone (Bp), respectively. Each panel depicts fits to the experimental data: the overall best fit is shown by the gray background, while the individual Gaussian curves belonging to a spectral feature are shown below. Colors are chosen to match the column labels in Table 1, where the peak center values (i.e., the eBEs) are provided. The theoretically calculated VIEs are plotted below each data set (cyan and orange bars correspond to the final singet and triplet states, respectively). Assignment of the radical anion spectral features using a comparison with UV–vis spectroscopy are depicted by a ruler in (A) and (C) following refs (9,60−63). The ruler indicates the low-lying triplet and singlet final states of the corresponding neutral aromatics. Each spectroscopic origin is referenced to S_0_ centered at the experimental position for the observed removal of the SOMO from the respective radical anion.

Figure 3A,C compares the PE spectra of the radical anions Np^–^ and Bp^–^ with those of the corresponding neutral aromatics Np and Bp (Figure 3B,D), all in THF. In each data set, the highest-intensity peak at VIE_liq_^THF^ = 8.45 ± 0.1 eV corresponds to the solvent’s HOMO of THF, as discussed above. For the neutrals, the lowest eBE peak should arise from ionizing the solute HOMO, giving rise to a spin doublet cation and a single additional assignable peak. This is the typical situation for PE spectra for closed-shell singlet species, like Np or indeed the solvent THF, as the only selection rule in PES is connecting configurations by the removal of a single electron. Thus, only spin doublet final states can arise from an initial singlet state. In the spectrum in Figure 3B, we only clearly see an additional peak for Np’s HOMO. Figure 3D shows the PE spectrum of a similarly concentrated Bp solution providing a spectrum with the same features as seen for neat THF. From these data, we can derive the solution phase VIE of Np, VIE_HOMO_^Np^ = 7.18 eV, which is almost 1 eV lower than the gas-phase one. The absence of a resolvable PE feature for the Bp HOMO might be expected based on the gas-phase VIE lying at 9.05 eV,^66^ some 0.6 eV higher than Np’s gas-phase IP.

The PE spectrum of the open-shell Np^–^ anion in Figure 3A has been determined from dissolving potassium in an Np-containing THF solution. The spectrum is recorded using He–II α radiation at 40.814 eV. In addition, we show in Figure S4 in the SI the PE spectrum recorded with the SOL^3^PES apparatus at BESSY with 123.464 eV photons when sodium/potassium alloy, sodium, or lithium metal is used as a reducing agent. We observe in all experimental spectra from BESSY II and FHI three weak but reproducible peaks with the lowest eBE peak around −2.7 eV. Unlike in fully occupied orbitals, photoemission from the singly occupied molecular orbital (SOMO), namely, the initial spin doublet anion state can only lead to a final spin singlet neutral configuration. Thus, the lowest-binding-energy peak corresponds to the lowest-energy configuration of Np, namely, the closed-shell singlet (S_0_) ground state and determines, therefore, the VDE_SOMO_^Np^–^^ = 2.73 eV.

For every other fully occupied orbital in the anion, we would expect two PE peaks separated by the singlet–triplet splitting for the neutral Np configuration associated with the removal of this electron. By removing an electron from the first fully occupied orbital, the final states will be the excited singlet (S_1_) and triplet (T_1_) states of neutral Np. In analogy with UV vis spectroscopy, the 0–0 origin for the T_1_–S_0_ transition lies at 2.64 eV in hexane solution as determined by oxygen-sensitized absorption.^61^ This suggests the assignment of the second lowest-lying peak (at −5.26 eV eBE) in Figure 3A as a detachment to T_1_. We can use the well-known optical spectra for solution phase Np, from its ground and lowest triplet states,^61^ to establish a ladder of expected final states. This is the ruler plotted in the top part of Figure 3A,C. This procedure suggests an assignment for each peak observed, as well as identifying when the photodetachment features is hidden under more intense features, e.g., due to ionizing THF. Comparisons to theoretical evaluation will provide further evidence to expand upon this assignment including the molecular orbitals involved.

The comparison of Np^–^ solution PE spectra provided in Section 3C in the SI shows two interesting additional details: first, while the two lowest eBE peaks are well reproduced when different spectrometers and radiation sources are used (Figure S3), in the region close to the first THF ionization feature, the third peak seems more pronounced when recorded with 40.8 eV radiation and a fitted peak center at −7.4 eV is closer to the onset of electrons ejected from THF than the experiment where *h*ν = 123 eV radiation is used. Given the possibility for multiple PE peaks in this region (see optical spectra ladder in Figure 3 and calculations below), our current understanding is that different PE angular distributions, as well as different energy-dependent cross sections for each of the overlapping detachment transition, are giving rise to the difference in appearance in this region. We note that the Helium lamp is unpolarized and the synchrotron radiation is linear horizontally polarized with respect to the electron detection direction (see Figure 1).

Second, it has been established that ion pairing takes place between aromatic anions and the alkali metal cations in solution, especially for solvents with modest static dielectric constants.^1^ It has been observed for Np^–^ in solution that the probability for contact ion pairing increases with the size of the alkali metal cation; it is therefore more likely for K^+^ to pair with Np^–^ than for Na^+^ and even less for Li^+^.^67^ However, general rules for ion pairing also consider the softness of the anion, based on polarizability.^68^ For example, for Bp^–^, the tendency for ion-pairing is reversed; it is observed that ion pairing is more likely with Li^+^ than for Na^+^ and even less for K^+^. The question we therefore explored was whether the binding energy is sensitive to the cation location in the first solvent shell, as is found for more polar solvents like water.^69^Figure S4 shows no peak shifts for solutions of Np^–^ with alkali metal cations generated from solid K and Na metal as well as NaK alloy. We do not yet have reliable data for Li where we might have expected to see a shift due to the solvent-separated ion pairing.

Figure 3C shows the PE spectrum of Bp^–^ in THF where we observe clear evidence for a more strongly bound excess electron in this radical anion. As Bp^–^ is a doublet, SOMO detachment leads to a single final neutral state (at eBE −3.55 eV), whereas transitions arising from fully occupied orbitals give a pair of transitions separated by the exchange interaction. The spacing and placement of these transitions given above the PE data in Figure 3C come from prior spectroscopic literature. The neutral Bp T_1_ energy relative to the ground state S_0_ (∼2.97 eV)^62^ is well known because of its widespread use as a triplet photosensitizer.^8^ The S_1_–T_1_ splitting is smaller for Bp (S_1_ at ∼3.65 eV in hexane)^63^ than Np and it appears that the broad and more intense peak observed in Figure 3C at eBE −7.15 eV does not resolve the singlet–triplet splitting. Our calculations below expand on the location of the higher-lying states.

In Section 3B in the SI, we provide a more detailed analysis where (1) we compare the relative advantages of synchrotron- versus lab-based valence band photoelectron (PE) measurements, (2) we examine the signal-to-noise ratio (SNR) and we consider the contrast relative to the background in the baseline region required to establish the energies for ejection from the weakly bound orbitals of e.g., the anion. We conclude that the absence of higher harmonic radiation with the helium lamp radiation source is an advantage providing a cleaner baseline in the lower-binding-energy region, while still providing high enough photon energies to avoid loss of spectral features in the low-energy tail from inelastic scattering.^70^

### 3.3 Simulation of the Np/Np^–^ and Bp/Bp^–^ Photoemission Spectra

With the range of experimental observations and an empirical assignment at hand, we will employ detailed electronic structure calculations for Np^–^ and Bp^–^ to fully interpret the measured spectral features in PE spectra depicted in Figure 3A,C. Note that the calculated VIEs are plotted as stick intensities below each experimental spectrum in Figure 3, while for the anion spectra in panels A and C, the singlet and triplet final states are indicated in blue and red, respectively.

**Figure 4:**
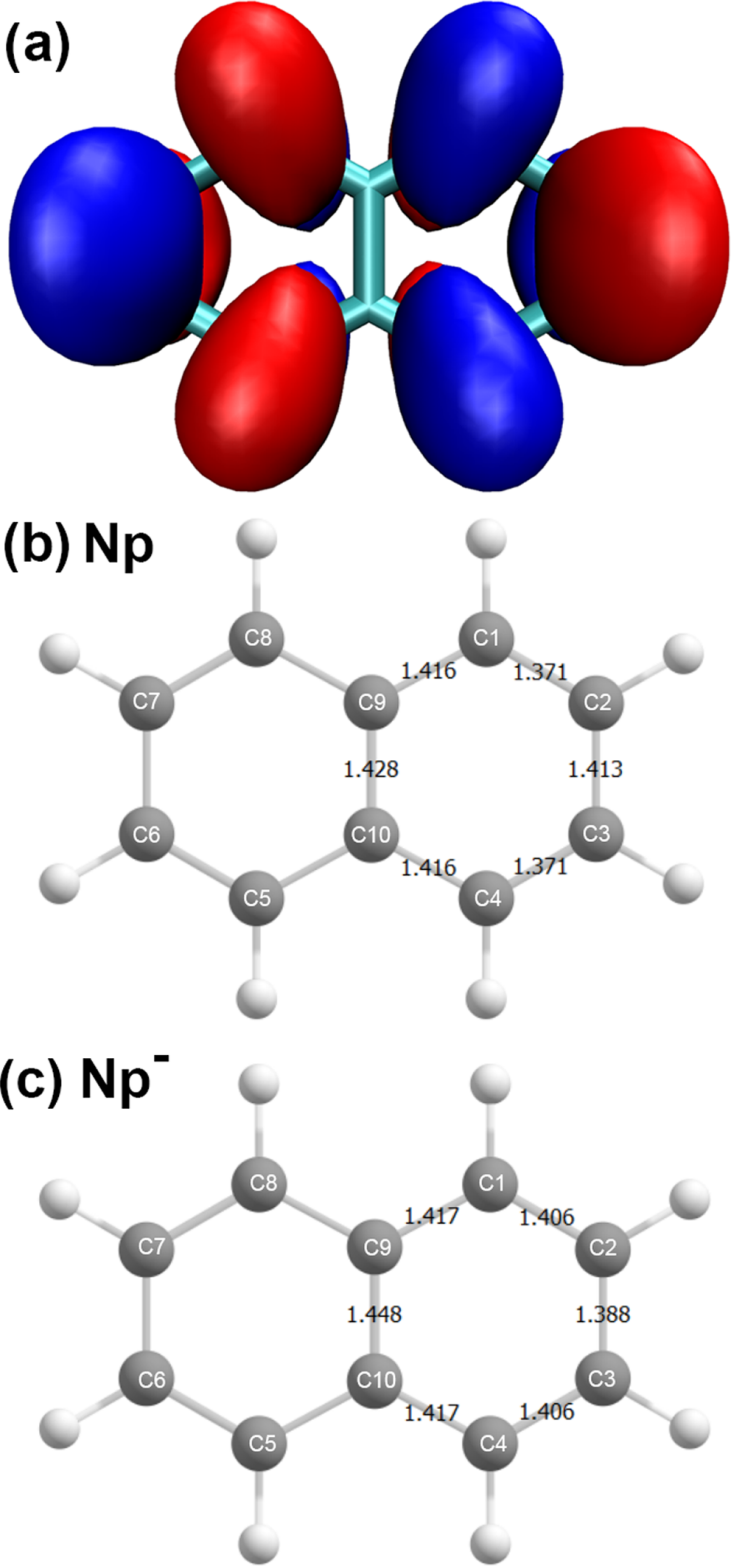
(a) SOMO of Np^–^, plotted with an isovalue of 0.025*a*_0_^–1.5^. (b, c) Theoretically predicted molecular geometries of neutral Np and its corresponding radical anion using the THF PCM model, respectively. A comparison of PCM structures with gas-phase structures can be found in the SI.

Np^–^ possesses a *D*_2*h*_ symmetry in its ground state with a calculated electron configuration of: ···π(b_1u_)^2^ (HOMO – 4), π(b_2g_)^2^ (HOMO – 3), π(b_3g_)^2^ (HOMO – 2), π(a_u_)^2^ (HOMO – 1), π(b_1u_)^2^ (HOMO), π*(b_2g_)^1^ (SOMO). The unpaired excess electron resides in the (b_2g_)^1^ singly occupied molecular orbital (SOMO) as shown in Figure 4A. A table with all π molecular orbitals of Np^–^ can be found in Figure S12 and Table S3 in the SI. The distribution of the spin density describing the delocalization of the excess electron is shown in Figure 7A in the SI. Ejection of the SOMO electron gives rise to S_0_ as depicted with a blue line at eBE –2.48 eV (Figure 3A). Removal of an electron from the doubly occupied b_1u_ (HOMO), results in the singlet (S_1_) or triplet (T_1_) state of neutral Np. Likewise, because electron removal from any lower-lying MO results in a pair of singlet and triplet final states, we have indicated these pairs in blue and red, respectively, in Figure 3A. Table 1 lists all calculated VIEs contributing to a measured spectral feature where the initial MO prior to emission is stated in brackets while the final state singlet and triplet character is indicated by the blue and red coloring in accordance with Figure 3A.

**Table 1:**
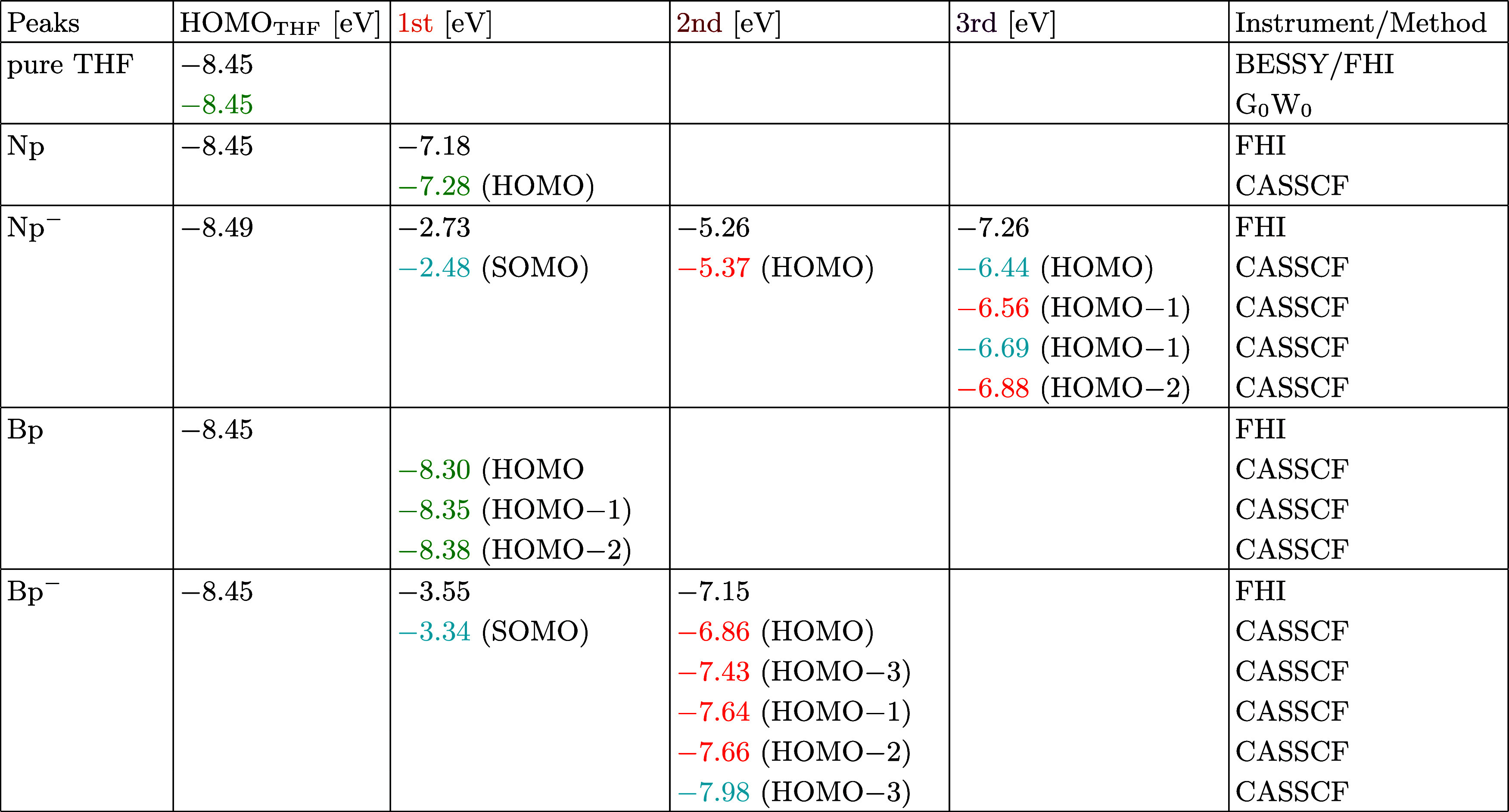
Overview of Electron Binding Energies (eBEs, While the Same, Positive Values Correspond to VDEs and VIEs) of the Studied Systems^a^

a The first column denotes the investigated system. The second to fifth columns present the spectral characteristics of the liquid HOMO of THF, followed by the third, second, and first peaks of the solute associated with solute orbitals. Experimentally determined eBEs are displayed in black, while theoretically calculated eBEs are denoted in blue for singlet, green for doublet, and red for triplet final states—all energies are given in eV. The MO contributing the most to the photoemission intensity is indicated within parentheses for each theoretical value. Note: the other MOs contributing as well as their orbital labeling are listed in detail in the SI. The last column states where the experiments have been conducted and the calculation method.

**Figure 5:**
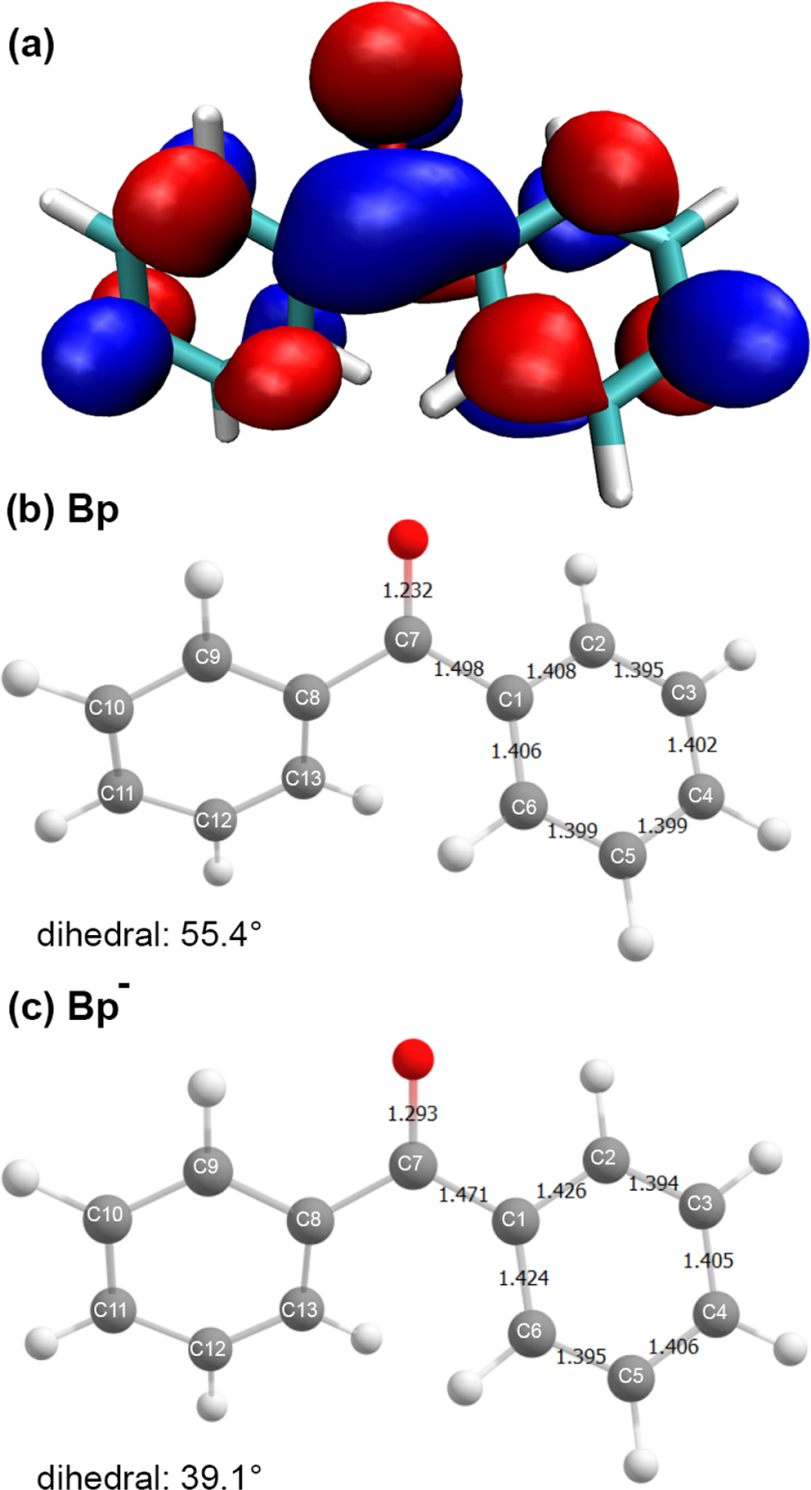
(a) SOMO of Bp^–^, plotted with an isovalue of 0.025*a*_0_^–1.5^. (b, c) Theoretically predicted molecular geometry of neutral Bp and its corresponding radical anion employing the THF PCM model. A comparison of PCM structures with gas-phase structures can be found in the SI.

Bp^–^ on the other hand has a *C*_2_ symmetry in its ground state with a calculated electron configuration of: ···π(1b)^2^ (HOMO – 6), π(1a)^2^ (HOMO – 5), π(2b)^2^ (HOMO – 4), π(3b)^2^ (HOMO – 3), π(2a)^2^ (HOMO – 2), π(4b)^2^ (HOMO – 1), π(3a)^2^ (HOMO), π*(5b)^1^ (SOMO). The unpaired excess electron resides in the (5b)^1^ SOMO shown in Figure 5A, while Figure S13 in the SI depicts all π MOs of Bp^–^. The spin density of Bp^–^ can be found in Figure S8B in the SI.

Ejection of the single electron from the SOMO results in the singlet ground state (S_0_) for neutral Bp; our calculations provide for Bp^–^ a VDE_SOMO_^Bp–^ = 3.34 eV, close to the measured peak VDE. Removal of an electron from the doubly occupied 3a (HOMO) results in either the lowest excited energy singlet (S_1_) or triplet (T_1_) state of neutral Bp and removal from any lower-lying MO results in further pairs of neutral singlet or triplet final states, which are indicated blue and red, respectively, in Figure 3C. In analogy to Np^–^, the lower part of Table 1 lists all calculated VIEs of Bp^–^ contributing to a measured spectral feature while the initial MO prior to emission is stated in brackets. Our calculations reveal that the second detachment feature for Bp^–^ located at eBE larger than −6 eV is quite complicated, arising from four triplets and one singlet final state. This accounts for the significantly larger area under this peak compared to the SOMO peak.

### 3.4 Gas–Liquid Solvation Shift

Table 2 summarizes the gas–liquid solvent shift Δ*G*_DFT_ estimated from electronic structure calculations using nonequilibrium PCM models. The VIEs of the neutral aromatics are predicted to be smaller compared to the gas phase by 0.90–0.95 eV for Np and 0.74–0.86 eV for Bp, respectively. This VIE decrease is similar (but smaller) compared to the one observed for THF itself. In simplest terms, the aromatic molecule being ionized is somewhat larger, and so the solvation energy for the final state cation is correspondingly reduced. On the other hand, for the anions, the effect of solvation on the VDE is much larger, ranging between 2.39 and 2.64 eV for Np^–^ and between 2.09 and 2.44 eV for Bp^–^. While an experimental value for the shift cannot be evaluated for Np, comparing our liquid phase VDE to ref (65) for Bp^–^ suggests an experimental shift of 2.6–2.9 eV. This difference and its implications on the stability of aromatic radical anions in solution will be discussed further below.

**Table 2:** Solvent-Induced Shifts Δ*G* of the HOMO Binding Energies (First Column) and Range of Solvent-Induced Shifts for All Calculated Binding Energies (Second Column) in eV Determined by Electronic Structure Calculations (DFT) for the Studied Systems of THF, Np, Np^–^, Bp, and Bp^–^a

	Δ*G*_DFT_	Δ*G*_DFT_ range	Δ*G*_BH_
THF	1.34	0.97–1.34	1.36
Np	0.91	0.90–0.95	0.94
Np^–^	2.47	2.39–2.64	2.66
Bp	0.74	0.74–0.86	0.78
Bp^–^	2.28	2.09–2.44	2.27

a For comparison, the last column lists the corresponding solvent shifts estimated using the classical Born–Haber cycle.

The Born–Haber cycle can be used to rationalize the Gibbs free energy of solvation Δ*G*_BH_ which are listed in the right column in Table 2. It accounts for the initial and final oxidation states of the species upon electron ionization that interact with different parts of the dielectric constant, hence the difference in the solvation shift for neutral and anionic species—exact details are given in Section 3D in the SI.

### 3.5 Estimating the Reduction Potentials and Thermodynamic Stability and Reactivity of Np^–^ and Bp^–^

While the VDE defines the stability of a species with respect to electron detachment upon absorption of ionizing radiation, the thermodynamic stability is determined by the ADE of the anion. Thus, the ADE of a species in solution defines which species form at equilibrium: in our case, the aromatic anions compete with the formation of other species such as solvated electrons (e_solv_^–^) or alkali metal anions (M^–^) in THF. Although, the formation of e_solv_^–^ and M^–^ have been demonstrated in THF by measurements of their absorption spectra, the concentration of both species on the time-scale of minutes to hours—and thus relevant for our experiment—is orders of magnitude smaller compared to the concentration of Np^–^ or Bp^–^ as is discussed in detail in Section 3G in the SI. We use the experimental PE spectra to evaluate the ADEs by estimating the onset at the low-binding-energy side of the lowest eBE spectral feature following the procedures described in ref (71). In Section 3F in the SI, we describe in detail how we infer ADE values from the measured PE spectra. Figure S5A–C in the SI shows the separately measured PE spectra of the solutes of Np^–^ and Bp^–^ and ferrocene (Fc) dissolved in THF, respectively. The approach of self-consistently measuring Fc also by liquid-jet spectroscopy avoids needing to apply literature estimates of Fc with respect to the vacuum level.

Thus, we determine directly the VIE_HOMO_^(Fc)^ = 5.8 eV for Fc in THF. Moreover, we estimate, relative to vacuum, ^(Np^–^)^ADE = 2.21 eV, ^(Bp^–^)^ADE = 2.80 eV, and ^(Fc)^ADE = 5.15 eV. Therefore, the PE spectra allow estimates of the reduction potentials, relative to a Ferrocene/THF standard as *E*_Np^–^_^0^ = −2.94 eV and *E*_Bp^–^_^0^ = −2.35 eV. These derived values agree well with those determined by cyclic voltammetry (CV), namely, ^CV^*E*_Np^–^_^0^ = −3.1 eV and ^CV^*E*_Bp^–^_^0^ = −2.3 eV, respectively.^1^ These results further provide confidence in our assignments and the solution identity within the liquid microjet.

## 4 Discussion

In the following, we first discuss the general nature of an excess electron associated with two quite different aromatic radical anions and the implications of the PE characterization from this work. Second, we reflect on the mechanism that leads to an increase in the binding energy of the excess electron upon solvation effectively resulting in the stabilization of the radical anions. Finally, we follow up on the solvent-induced stability and its implications on the reactivity of radical anions as reactive intermediates within the Birch reduction process.

### 4.1 The Electronic Structure and Nature of the Solution Phase Radical Anions

Neither Np^–^ nor the benzene radical anion (Bz^–^) are stable species in the gas phase. Experimentally, however, the technique of electron transmission spectroscopy allows one to infer the vertical EAs of unbound resonant states; thus, for Bz^–^ and Np^–^, VEA_Bz^–^_ = −1.15 and −4.85 eV and VEA_Np^–^_ = −0.19, –0.90, –1.67, –3.37, –4.72 eV have been determined, respectively.^72^ In contrast, the anthracene radical anion (Ant^–^) already possesses a positive VEA_Ant_ = 0.53 eV, thus being the smallest unsubstituted aromatic hydrocarbon forming a stable radical anion in the gas phase.^73^ Similarly, Bp^–^ is a stable compound already in the gas phase with a VDE of 0.91 eV^65^ which is in good agreement with our theoretical computed value of VDE 1.07 eV. The difference in VIEs between Bp and Np is largely due to increased localization of the excess charge on a carbonyl antibonding orbital in Bp (see Figure 5A), rather than in the π* systems of the phenyl rings (see below).

For the polyacenes, however, we then ask how the extra electron becomes bound upon solvation. For Np^–^, more than 2.5 eV of vertical stabilization for the excess electron is gained by immersion in the THF solvent. Does the environment simply provide electrostatic stabilization to the extra electron that is localized in a rather similar π* LUMO as expected from gas-phase electronic structure calculations? Or does the formation of a solvation shell extend the molecular entity such that it enables the wave function of the excess electron to delocalize into the solvent leading to a more stable molecular structure of the otherwise unstable anionic species? The latter would clearly have an impact on the reactivity of the radical anion, making it a much softer nucleophile. One might imagine the latter description of the excess electron as a hybrid between a solvated electron occupying interstitial space in the liquid and an orbital localized above and below the aromatic plane. We note that THF is known to have considerable excess space in the solvent network which is used to support excess electron density when excess electrons are generated by photodetachment or photolytic or radiolytic ionization.^13,59,74,75^ A related question is whether the unoccupied orbitals in the solvent play a role by accepting part of the electron density, as occurs with excess electrons in ammonia and water ices, as implicated by EPR spectroscopy.^19,76−78^

Beyond determining energetic properties like the VDE and the reduction potential, we can compare our electronic structure calculations to molecular geometry and vibrational frequency data and relate them to other polyacenes and their radical anions. The addition of an electron to the LUMO in *D*_2*h*_ aromatics has long been known to activate the bond alternation pattern in the C–C bond lengths in the molecular frame. For Np, electronic excitation of the neutral molecule along the π* ← π transition increases the bond order in C2–C3 and C6–C7 (shortening by 0.05 Å),^79^ while decreasing in C1–C2 (and symmetry-related C–C bonds) as well as the central C–C bond (elongating by 0.06 and 0.04 Å, respectively, see Figure 4 for naphthalene carbon numbering). This can be understood by inspection of the nodal pattern in the HOMO and LUMO orbitals shown in Figure S12 in the SI. The change in the bonding pattern is sufficient to give a long Franck–Condon progression in the electronic absorption spectrum in the totally symmetric C–C framework stretching modes.^80,81^

Upon removal of a SOMO π* electron from the molecular anion, one would expect an oppositely signed bond alternation change, but not as dramatic in magnitude because an electron has not been changed in the π HOMO. Gas-phase high-resolution PE spectra of anthracene radical anion (Ant^–^) report^82^ detachment to the singlet and triplet states on an equal footing, as is seen in our work. In their PE spectra, the origin is the most intense peak, but there is again vibrational activity in the totally symmetric stretch modes. Activity is observed in the totally symmetric framework vibrations (assigned as ν_6_ = 1267 cm^–1^ and ν_7_ = 1416 cm^–1^ in ref (82)), while simulations of the Franck–Condon progressions are consistent with calculations that show a bond alternation change of 0.022 Å with a pattern consistent (lengthening C2–C3, shortening C1–C2, etc.) with the qualitative expectations outlined above. The mean absolute C–C change is about half of that connected with the π* ← π excitation from S_1_ ← S_0_.^79^ The PE spectrum also reveals a lesser degree of C–C frame activity in ν_6_ and ν_7_ induced on the removal of the π electron to reach T_1_. Moving to the solution phase, Juneau et al.^83^ report a significant (30–45 cm^–1^) drop in the 1260 and 1400 cm^–1^ (ν_6_ and ν_7_) vibrational frequencies for Ant^–^ compared to Ant in the measured resonance Raman spectra, consistent with the addition of an electron to a characteristic π* orbital.

While we do not observe vibrational progressions in the liquid phase PES because of solvent-induced broadening, our calculations present a picture matching the qualitative pattern described above for the anthracene radical anion, where calculations and experiment provide a consistent description. Figure 4B,C shows a computationally predicted bond alternation in Np and Np^–^, respectively. Addition of the excess electron results in shrinking of ∼0.025 Å in the C2–C3 and C7–C8 bonds and elongating most notably of C1–C2 (and symmetry equivalent bonds) by 0.03 Å as well as the C9–C10 central bond by 0.02 Å. Comparison with the X-ray crystallography data of the [C_10_H_8_^•–^][Na^+^(diglyme)_2_] crystals grown from diglyme solutions where Np had been reduced by sodium,^84^ reveal very similar bond length changes in the Np^–^ anion: the outer pair of the C2–C3 and C7–C8 bonds contract by ∼0.03 Å, while the other nine C–C bonds elongate by 0.02–0.03 Å.^84−86^ Although the environment for the Np^–^ is very different in the two latter cases—namely, an unstructured liquid versus a periodic crystalline structure—the strong similarities in the bond length changes show the influence of the excess electron density onto the Np molecular structure. This result strengthens our confidence that LJ-PES in conjunction with quantum chemical calculations enables also to acquire structural information.

One can anticipate that if an isolated molecular ion picture applies to Np^–^ in THF, some of the width and peak shape observed in the D_0_ ← S_0_ PE band arises from Franck–Condon activity in this framework distortion. Because the calculations employ a polarizable continuum model, they cannot address whether the excess electron density is entirely localized in the π* orbital. But the continuum model does correctly predict the magnitude in the VDE shift from vacuum into THF and it is hard to see how this would happen if we had an entirely incorrect binding motif for the electron. More detailed multireference computations with explicit THF solvent molecules are too costly and methodologically demanding at the current time. But, as a further test that the excess electron is localized on the solute molecule and does not spread to the solvent molecules, we put one explicit THF molecule in the CAS-SCF calculations. This did not lead to any significant change in the spatial distribution of the spin densities on either anion (see Figure S9 in the SI for spin densities with an explicit solvent molecule).

Let us consider the bond length changes one step further by reviewing vibrational spectroscopy of Np^–^ anion in THF.^87−89^ Just as for Ant^–^, a downshift in the three a_g_ C–C stretching modes (1579, 1460, and 1379 cm^–1^ in the neutral) by about 30–40 cm^–1^ is suggestive that, in solution, the electron density is added to an antibonding orbital changing the π bond order and thus the force constants relative to neutral Np. Perhaps even more convincing evidence comes from the observation of high IR band intensities for Np^–^ assignable to large charge flux between Np rings via motion along the bond alternation mode. We conclude that the calculations presented here for the anion are representative of the true solution phase where significant localization of the excess electron occurs into the π* orbital of Np.

Shifting now to Bp^–^, Figure 5B,C depicts the calculated distortions of the aromatic ring on adding an electron to Bp. The largest effects arise around the carbonyl moiety where most of the excess charge in Bp^–^ is predicted to reside. This change in bonding around the carbonyl group resulting from the addition of an electron to neutral Bp (a longer C–O bond as well as shorter C1–C7(C8) bonds) is accompanied by a significant change in the twist between the two phenyls.

The Bp structure exhibits a dihedral angle between the two rings of 54° in the gas phase with a similar value of 55° in a dielectric matching THF. Note that these values align closely with the dihedral angle of approximately 56° obtained from X-ray crystallography of crystalline Bp, as observed experimentally.^90^ In contrast, the optimized structure of the Bp^–^ displayed a significantly more planar conformation, with dihedral angles of 36° in the gas phase and 39° upon solvation in liquid THF. This increased planarization of Bp is also in line with experimentally measured Raman spectra^83^ that supported the shift to a more planar structure and the partial localization of the excess electron density onto the carbonyl functional moiety.

### 4.2 Progressive Stabilization by Solvent

There have been numerous experimental and theoretical studies in the past that investigated VEA shifts of the excess electron upon microsolvation pointing to increased stability of the radical anion in solution.^1,7,10^ However, this is the first measurement of the impact of complete bulk solvation on the energetics. The addition of methanol (MeOH) ligands to Bp^–^(MeOH)_*n*_ in the gas phase has been shown to shift its EA_*n*=0_ = 0.91 eV, to EA_*n*=1_ = 1.29 eV, EA_*n*=2_ = 1.65 eV, EA_*n*=3_ = 1.74 eV.^65^ In a recent paper, Verlet and co-workers observe that upon solvation of the anthracene anion by water, on average each of the first shell waters increases the VDE by ∼0.2 eV.^91^ They also observe the singlet–triplet splitting, 1.93 eV, does not change on solvation. Similar stabilization takes place also for species unstable in the gas phase like Np^–^(Bz)_*n*_ or Np^–^(H_2_O)_*n*_, which become stable by attaching a single (*n* = 1) ligand molecule evidenced by measured positive EA being EA_Np^–^(Bz)_1__ = 0.03 eV^7^ and EA_Np^–^(H_2_O)_1__ = 0.11 eV.^10^

There are numerous molecular cluster studies investigating the progressive solvation and increasing stabilization of neutral and charged species ranging from e.g., solvated electrons in increasingly sized clusters of water, MeOH, THF, etc.^92^ up to larger species such as anionic nucleobases in increasingly sized water clusters.^93^ These cluster studies show practically the same tendencies as we observe for the bulk solvation of Np and Bp and their anions (compare Table 2): first, the solvent-induced shift is larger for the anionic than for the neutral species which results from the stronger charge ↔ dipole versus the induced-dipole ↔ dipole interaction and, second, the solvent-induced shift is larger for Np where an induced-dipole ↔ dipole interaction competes with a dipole ↔ dipole interaction in Bp.

Kostal et al.^28^ investigated in a theoretical study step-by-step the whole solvation process from the isolated gas-phase species all the way to the liquid bulk. It was shown there that the Bz^–^ requires between 7 and 10 ammonia molecules to reach negative VDEs for the excess electron, i.e., to stabilize the species. Also, it takes hundreds of solvent molecules to converge to the VDE bulk value. A related study of the same system in bulk liquid ammonia demonstrates that while the excess electron is well localized on the benzene moiety (leading also to its Jahn–Teller distortions), the electronic stability of Bz^–^ results dominantly from interactions with the solvent ammonia molecules providing dielectric stabilization.^27^

### 4.3 Mechanism of Excess Charge Stabilization

From the above considerations we argue that for both investigated molecules, it is the change in the dielectric constant from the presence of solvent that provides the main mechanism to stabilize the excess electron and this dominant effect is being well recovered by the PCM calculations. This conclusion is independently supported by applying the simple Born–Haber cycle model—see Table 2 and the detailed Section 3D in the SI for a quantitative comparison—which we envision to be operative in analogy to the recent study of sequential solvation Bz^–^ in ammonia.^28^

The fact that the stabilization mechanism for radical anions is dominantly an electrostatic mean field effect, implies two characteristic properties of aromatic radical anions in solution: first, the density of states and thus the shape of the SOMO (as well as the lower-lying MOs) should remain largely unaffected by solvation. Second, the fact that a simple Born model appears to be well suited to describe all MO shifts for the radical anion according simply to the dielectric constant, opens up the possibility of quantitatively exploring the tuning of the stability and reactivity of radical anions, solvent-dependent, as reflected in the respective ADE value. ADE can, in turn, be estimated from the measured VDE peak positions and widths of the spectrally isolated SOMO band. Thus, within the range allowed by practical solvents, a liquid-jet PE measurement can verify VDE tuning by considering the characteristic dielectric constant.

For example, such tuning could be achieved on the one hand, e.g., using acetonitrile (ε_r_ = 37.5) and, on the other hand, for example, 1,4-dioxane (ε_r_ = 2.2). Binary mixtures of two solvents can be produced at any ratio allowing to continuously adjust the dielectric constant as demonstrated in refs (94,95). Assuming solvent mixing is happening also at the nanoscale, the VDEs of Np^–^ could be tuned between 3.05 and 1.81 eV while those of Bp^–^ could be tuned between 3.83 and 2.76 eV using binary mixtures of different ratios of acetonitrile and 1,4-dioxane. This enables an enormous tuning range of 1.24 eV (28.6 kcal/mol) and 1.07 eV (24.7 kcal/mol) for Np^–^ and Bp^–^, respectively. Acetonitrile may be a better-suited solvent to increase the stability of radical anion intermediate, but it also stabilizes the solvated electron.^96^ 1,4-Dioxane on the other hand increases the reactivity, but only moderate concentrations of the radical anion species can be achieved in this unpolar solvent.

Researchers on aromatic carbanion intermediates have long worked in a narrow range of ethereal solvents, and have found that the countercation provides the most subtle and local electrostatic control on tuning the chemistry, as well as exploiting crown ethers to sequester alkali ions. Our measurements have not yet explored this tunability, but with the binding energy precision now possible using cutoff-based calibration, we can anticipate that such information will be forthcoming. For the time being, we note that given prior literature suggests that Np^–^ exists in contact ion pairs with K^+^, the VDE and ADEs reported here therefore most likely correspond to the energetics from the ion pair, rather than the free Np^–^.

### 4.4 Reactivity in the Context of Birch Chemistry

**Figure 6:**
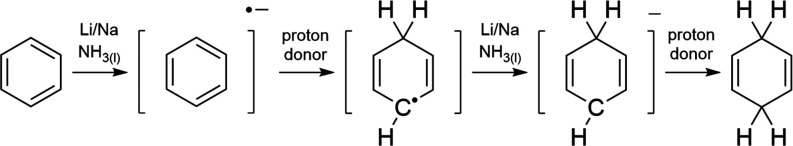
Schematic depiction of the two main reaction steps of the Birch reduction of benzene in liquid ammonia.

The above considerations influence the chemical reaction dynamics which we now discuss in the context of Birch reduction. Mechanistically, Birch chemistry proceeds in multiple steps involving sequential addition of electrons and protons (the latter typically provided by a suitable alcohol), eventually leading to regioselective reduction of a wide variety of aromatic compounds—as Figure 6 illustrates for the iconic example of the conversion of benzene into 1,4-cyclohexadiene.^2^ The present study is relevant for the first step of this process, namely, the formation of the aromatic radical anions, and the stability of this species. Based on our detailed investigations concerning the solvation process, we draw here attention to the solvent influence given primarily by its characteristic dielectric constant.

The classic Birch reduction is conducted in liquid ammonia^2^ but because ammonia is liquid only below −33 °C refrigeration of the reaction mixture is required. Liquid ammonia also strongly stabilizes solvated electrons and dielectrons,^19^ competing with the formation of aromatic radical anions. Indeed, in liquid ammonia at higher electron concentrations, it is more favorable to form solvated dielectrons than benzene radical anions.^97^ The choice of other polar media such as THF or acetonitrile leads to lesser stabilization of the solvated electrons which persist only on micro- to millisecond time scales.^13,96^ At the same time, aromatic radical anions can be formed in these solutions at up to molar concentrations. Concerning the first step of Birch reduction, one is seeking a solvent environment providing the best compromise between stability and reactivity of the aromatic radical anions.

Recent studies explored various solvents with the aim of realizing Birch reduction at room temperature.^5^ An important conclusion was that Birch reaction could be achieved using a binary solution mixture; for an optimized overall yield, an ether host along with an amine cosolvent should be present. For example, using THF as solvent environment and mixing in molar equivalents of both ethylenediamine (EDA) and lithium to substituted benzene compounds (e.g., benzoic acid) enabled the authors to achieve higher product yields compared to using pure EDA, as if the reaction had been carried out in liquid ammonia.^5^ Note that for a pure THF solution, no Birch reduction was observed^5^ as most likely this hinders the rate-determining step which is the proton transfer to the aromatic radical anion (Figure 6).

Mixing THF (ε = 7.42^58^) with EDA (ε = 15.5^98^) over the full range of mole fraction (changing the overall solution dielectric constant) would allow the eBE to be tuned over a range of 5 and 4 kcal/mol in Np^–^ and Bp^–^, respectively. Under the conditions given for the optimized Birch reduction of Np^5^ (see Section 3E in the SI for details), the intermediate Np^–^ is more reducing by 0.13 eV (3 kcal/mol) using a ternary solution of THF and EDA with tert-butanol as proton donor compared to a pure EDA/tert-butanol solution. It is not clear yet how the change of the solvent dielectric constant influences other steps of the Birch reduction and how this results in a final increase of the product yield^5^ but the current work provides a clear pathway to quantify and predict how to tune the reactivity of the first intermediate. It should help in the quest to identify and tailor the optimal combination of (i) the solvent environment, (ii) the alkali metal, and (iii) the most suitable proton donor to optimize the conditions for the Birch reduction of a particular aromatic compound. This, however, remains a task for future studies going beyond the present investigation characterizing the aromatic radical anion as the first intermediate of the Birch reduction.

## 5 Conclusions

In this study, we conducted the first quantitative investigation of the valence electronic structure of aromatic radical anions in the solution phase employing a combination of LJ-PES measurements and quantum chemical calculations. Our results provide a detailed understanding of the stabilization mechanism of the radical anion species upon solvation which turns out to be dominantly an electrostatic mean field effect. Thus, the change of the dielectric environment stabilizes the excess electron by ∼3 eV localizing it on the aromatic in a valence π* orbital which was unbound in the gas phase. There is no evidence for the excess electron delocalizing into the solvent. Additionally, we find very good agreement of the redox potentials extracted from the present LJ-PES measurements of Np^–^ and Bp^–^ in THF with electrochemical data from CV measurements.^1^ The present work allowed us also to relate directly to the chemical reactivity of these radical anions in solution in the context of Birch reduction in liquid ammonia as well as in room-temperature solvents.

## Data Availability

The data that support the findings of this study are openly available in Zenodo at: 10.5281/zenodo.10515804.

## Supporting Information Available

Additional details on the experimental procedures, binding energy calibration of THF, conformer resolution of THF EDOS, data analysis for radical anions measurements, and comparison of synchrotron and lab-based measurements as well as comparison of different cationic species in radical anion solutions; more detailed comment on the solvation free energy, spin densities of the studied radical anion species, and their spin densities in the presence of one explicit solvent molecule; and geometries of the radical anions and neutral species in both the gas phase and PCM, along with insights into molecular orbitals and leading determinants of the CAS calculations (PDF)
