# Supplementary material for: Stability and Reactivity of Aromatic Radical Anions in Solution with Relevance to Birch Reduction
Source: J Am Chem Soc. 2024 Feb 16;146(12):8043–57. doi: 10.1021/jacs.3c11655 (PMC10979400; doi:10.1021/jacs.3c11655)
Supplement: Supplementary file 1 — ja3c11655_si_001.pdf [file ja3c11655_si_001.pdf]

# Supplementary Information: Stability and Reactivity of Aromatic Radical Anions in Solution with Relevance to Birch Reduction

Tatiana Nemirovich†,[1, a)] Brandon Young†,[2] Krystof Brezina†,[1] Philip E. Mason,[1] Robert Seidel,[3] Dominik Stemer,[4] Bernd Winter,[4] Pavel Jungwirth,[1] Stephen E. Bradforth*,[2, b)] and H. Christian Schewe*[1, 5, c)]

[1)] *Institute of Organic Chemistry and Biochemistry of the Czech Academy of Sciences, Flemingovo nám. 2, 166 10 Prague 6, Czech Republic*

[2)] *Department of Chemistry, University of Southern California, Los Angeles, California 90089, United States*

[3)] *Helmholtz-Zentrum Berlin für Materialien und Energie, Hahn-Meitner-Platz 1, 14109 Berlin, Germany*

[4)] *Fritz-Haber-Institut der Max-Planck-Gesellschaft, Faradayweg 4-6, 14195 Berlin, Germany*

[5)] *J. Heyrovský Institute of Physical Chemistry, Czech Academy of Sciences, Dolejškova 3, 18223 Prague, Czech Republic*

(Dated: 17 January 2024)

## CONTENTS

**1. Experimental procedures: solution preparation, transfer and jet-generation** 1

**2. THF solvent characterization** 2
- A. Electronic density of states 2
- B. Gas- and liquid-phase binding energy calibration of THF 2
- C. Conformer resolution of THF gas-phase EDOS 2

**3. Solute characterization in THF** 3
- A. Data-analysis and data-fitting 3
- B. Comparing synchrotron and lab-based valence band photoelectron spectroscopy measurements 3
- C. Comparison of different cationic species in solutions of $Np^-$ in THF 5
- D. Estimation of the solvation free energy: PCM calculations and Born-Haber cycle 5
- E. Estimating VDE and reduction potential shifts as a function of the solvent's dielectric using the Born model 6
- F. Estimating the reduction potentials and thermodynamic stability of $Np^-$ and $Bp^-$ in THF 6
- G. Formation of different solute species in THF 7
- H. Fit of experimental spectrum using theoretical binding energies as input 8

**4. Theoretical procedures** 10
- A. Comments on the theoretical methods 10
- B. Spin density of $Np^-$ and $Bp^-$ 10
- C. Does the spin density "leak" to the nearest solvent molecules? 10
- D. Geometries of the radical anions and corresponding neutrals considered in this work 10
- E. Molecular orbitals of naphthalene and benzophenone radical anions and leading determinants for each of the CAS calculations 13

**References** 16

## 1. EXPERIMENTAL PROCEDURES: SOLUTION PREPARATION, TRANSFER AND JET-GENERATION

The purification of tetrahydrofuran (THF) was performed using a distillation unit typical for organic chemistry laboratories. In this process, a large round-bottom flask was filled with 1 L of THF (Lachner 99%), 12 g of Benzophenone (Bp)(Merck), together with an excess of slices of Potassium metal (Sigma Aldrich) under an Argon atmosphere.[1] The formation of benzophenone radical anions ($Bp^-$ ) in THF reacted with residual impurities - especially water and oxygen. After some time, the THF solution turned blue which indicated that $Bp^-$x had reacted with all undesired species in THF while after some days the solution even turned purple indicating the formation of Bp di-anions ($Bp^{2-}$).[1] The THF was brought to reflux at an operating temperature of 80°C where the evaporated THF was condensed by a water-cooled condenser and collected in the distillation head. The distilled THF was then drained directly into solution flasks via a release port, thus avoiding atmospheric contact.

Solution procedure: A flask with a Young's valve was introduced into a glove box under nitrogen, filled with the solid reagents, sealed, and then transported to the THF distillation unit. The flask was then filled with distilled THF, while still under an inert atmosphere, and stirred to elicit the reaction. After some time the solution turned dark green or blue in color indicating the formation of the naphthalene radical anion ($Np^-$) or $Bp^-$, respectively. In later experiments at FHI, the green solution was filtered

---

a) †These authors contributed equally.
b) Electronic mail: stephen.bradforth@usc.edu
c) Electronic mail: hanns_christian.schewe@uochb.cas.cz

through a polyethylene (PE) screw-top column tube that had a series of PE filter discs. This extra filtering step turned out to be crucial to having a liquid micro-jet (LJ) run longer than 20 minutes in the vacuum chamber. The PE column tube was then charged with a nitrogen head-space, sealed off, moved and adjoint to the cryostat unit mounted at either the $SOL^3PES$ instrument at BESSY II synchrotron or at the EASI instrument at FHI.

The reservoir in the cryostat unit was evacuated first to ensure that there was a pressure differential between the PE column tube and the sample chamber. Once ready, the column tube was opened to the sample chamber via a three-way stopcock. After solution transfer an argon pressure of about 1-2 bar relative to vacuum was applied to the head-space of the cryostat in order to initiate the LJ to run constantly while photo-electron (PE) spectra were recorded.

THF liquid jets pose an interesting design challenge due to their inherent instability after the laminar region. It was commonly observed that 1-2 millimeters after the laminar region, the jet would either curve laterally or sputter into droplets causing an increase in the pressure of the sample chamber. Since the THF mainly evaporated rather than being frozen out in the downstream cryotrap we seldomly observed the formation of dendroid-like structures close to the nozzle orifice, the skimmer of the hemispherical electron analyzer, or the capillary of the light source.

After all experimental runs, we cleaned the transfer columns of the cryostat unit as well as the $SOL^3PES$ or EASI instruments applying the appropriate precautions especially when handling alkali-metal.

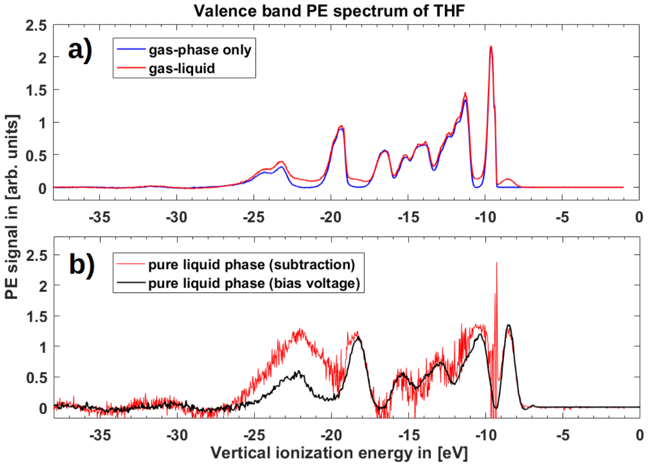


Figure S 1. VB spectra of THF: panel a) measurements gas-phase (blue) and gas-liquid-phase spectra of THF. Panel b) pure liquid-phase spectra - experiments were conducted at BESSY II synchrotron abusing $SOL^3PES$ instrument.

| peak | $BE^{lit}_{gas}$ [eV] | $BE^{meas}_{gas}$ [eV] | $BE^{meas}_{liq}$ [eV] | $\Delta BE_{gas\text{-}liq}$ [eV] |
|---|---|---|---|---|
| 12a’ | -9.73 | -9.62 | -8.45 | 1.28 |
| 11a/11a’ | -11.37 | -11.28 | -10.07 | 1.27 |
| 10a/8a” | -11.99 | -11.89 | -10.61 | 1.18 |
| 8b/7a” | -12.40 | -12.47 | -11.08 | 1.24 |
| 9a/10a’ | -12.91 | -12.95 | -11.57 | 1.21 |
| 7b/6a” | -13.87 | -13.81 | -12.52 | 1.21 |
| 8a/9a’ | -14.54 | -14.44 | -13.24 | 1.26 |
| 6b/5a” | -15.20 | -15.25 | -13.94 | 1.31 |
| 7a/8a’ | -16.35 | -16.31 | -14.97 | 1.18 |
| 5b/7a’ | -16.69 | -16.79 | -15.51 | 1.38 |
| 6a/4a” | -18.96 | -19.26 | -17.72 | 1.32 |
| 4b/6a’ | -19.24 | -19.54 | -18.26 | 1.58 |
| mean | | 0.11 | | 1.285 |

Table S 1. Gas-liquid solvation shift of individual spectral features of THF in THF. The ’mean’ value is the mean deviation between the gas phase literature values and the measured gas or liquid phase values.

## 2. THF SOLVENT CHARACTERIZATION

### A. Electronic density of states

To model the electronic density of states, we use the eigenvalue-self-consistent version[2] of $G_0W_0$ (evGW), which provides iterative corrections building on the self-consistency of the energy eigenvalues only, without changing the corresponding one-electron states. The calculation was accelerated using the resolution of identity approach with the RI-TZ auxiliary basis and further relied on the Padé approximation to analytically continue the self-energy on the real frequency axis and the Newton-Raphson fixed point iteration to numerically solve the relevant algebraic equations. The evGW energy corrections were calculated for all occupied orbitals and additional 10 virtual orbitals. The resulting corrected quasiparticle energies were binned into a histogram with a 0.10 eV bin width over the range of $-35$ to $-5$ eV to represent the electronic density of states (EDOS).

### B. Gas- and liquid-phase binding energy calibration of THF

Panel a) of Figure 1 depicts the measured gas-and-liquid-phase spectrum (red line) and gas phase spectrum (blue line) from a pure liquid THF microjet with a salt concentration of 0.1 mol/L $TBA^+PF_6^-$.

### C. Conformer resolution of THF gas-phase EDOS

In order to resolve the EDOS presented in Figure 1 of the main text into the contributions of each equilibrium conformer and its surrounding thermal population,

we employ an unsupervised machine learning technique. Specifically, we use the $k$-means clustering method, as implemented in the Python Scikit-learn library,[3] to divide the set of configurations visited during the AIMD simulation into subsets corresponding to each potential energy basin. To reach this goal, we choose $k = 4$, because the potential energy surface has 4 minima: two corresponding to $C_s$ symmetry and two to $C_2$ symmetry. Note that within both pairs, the structures are chemically fully equivalent, but they all are represented by unique points in the configurational space due to different atom indexing. The obtained cluster centroids are shown in Figure 2 and correspond very well to the optimal conformer geometries with appropriate symmetries. The EDOS resolved to each conformer was then obtained by binning quasi-particle energies only of the subset of configurations that fall to the given cluster. These are shown in Figure 2 as blue lines. A comparison with the full EDOS (Figure 2 in gray, taken over the whole set of configurations as discussed in the main text) does not point to any meaningful deviations and allows us to conclude that all THF conformers give rise to a very similar thermal density of states with negligible differences within the available statistics.

## 3. SOLUTE CHARACTERIZATION IN THF

### A. Data-analysis and data-fitting

The PE spectra were measured by acquiring individual sweeps where the PE signal was recorded as a function of the kinetic energy. The acquired PE spectral sweeps were averaged. In order to determine the vertical ionization energies (VIEs), i.e. the binding energies (BEs) of the spectral features accurately we applied established procedures described in the references 4,5. All spectral features of the valence band as well as the low kinetic energy cutoff (see e.g. Figure 1 c) of the main manuscript) were fit using the same methodology within MATLAB: a non-linear regression routine was applied to fit a designed fit-function to the data. This overall fit-function consists of a number of $i$ Gaussian functions ($a_i$exp[-($E_{\rm bind}$ - $E_i^{\rm SF}$)/$c_i$]) each representing a spectral feature (SF) of the valence band. Thus, the output of the overall fit function contains the sum of all spectral features while their individual contributions are given by the amplitude $a_i$, the $\mathrm{VIE}_i^{\rm SF}$, and the width $c_i$ of the spectral feature.

### B. Comparing synchrotron and lab-based valence band photoelectron spectroscopy measurements

To test for reproducibility of the recorded data, PE spectra of $Np^-$ in THF were recorded multiple times, using different spectrometers and light sources. Figure 3 compares spectra recorded with the linear polarized synchotron radiation (BESSY, upper panel) to that recorded with unpolarized Helium lamp radiation on the EASI spectrometer at FHI (lower panel), along with overall fits to data. In both datasets, the high binding energies features from THF photoemission are not shown but the peak heights of the features arising from $Np^-$ are shown on a scale relative to the THF HOMO peak. In both cases, three distinct features are evident with the two lowest binding energy features in good agreement as shown by the dropped vertical lines. The comparison of the peak intensities of each individual spectral feature reveals differences: while for the BESSY measurements all three peaks show the approximately the same intensity, the FHI peak intensities clearly differ.

Interestingly, the peak at the highest binding energy of the three peaks is noticeably more intense in the lower panel compared to both the lower energy peaks and in comparison to the synchrotron data and its peak center is shifted to higher binding energy by about 0.5 eV. Notably, the low binding energy onset of the third peak is practically the same at ∼-5.5 eV in both measurements.

These differences may be due to different transmission probabilities of the lens tables used of the different hemispherical analyzers (Scienta Hipp2 at BESSY and Scienta Hipp3 at FHI) with respect to the different electron kinetic energies. But, the fact that the highest energy feature likely originates from detachment to several final states (see Figure 3 and Figure 4 and assignments in the main text) provides an alternate explanation of this effect. As noted, the ionizing radiation was linearly polarized at $SOL^3PES$ while at EASI the light was unpolarized. Related, the experimental geometries of the $SOL^3PES$ and EASI spectrometers are also different. The position of the skimmer and thus the detection direction with the hemi-spherical analyser was at 90°at $SOL^3PES$ compared to the 70°at EASI – see Figure 1 of the main text. Therefore any differences in the photoelectron angular distributions between peaks can lead to different relative peak intensities. For the third peak, this effect can also emphasize different sub-components underlying a broad peak composed of multiple final states. For example, on the lower binding energy side, detachment to $S_1$ final neutral state may arguably have a similar angular distribution to the peak assigned to the $T_1$ final state near -5.5 eV as they arise from electron removal from the same orbital. But on the higher binding energy side of this feature, detachment to $T_2$ and $S_2$ final states both contribute, and these involve removal from a different occupied molecular orbital that may detach with a different PAD. Finally, we note that one possibility that cannot be completely ruled out is residual neutral naphthalene contributing to the third peak, although excess alkali metal was used in all experiments.

To compare the relative efficacy of synchrotron- versus lab- based valence-band (VB) photoelectron (PE) measurements, we have made a more detailed comparison of the quality of spectra by examining the background level and the signal-to-noise ratio (S/N). PE spectra of neutral naphthalene in THF (not shown) were also recorded

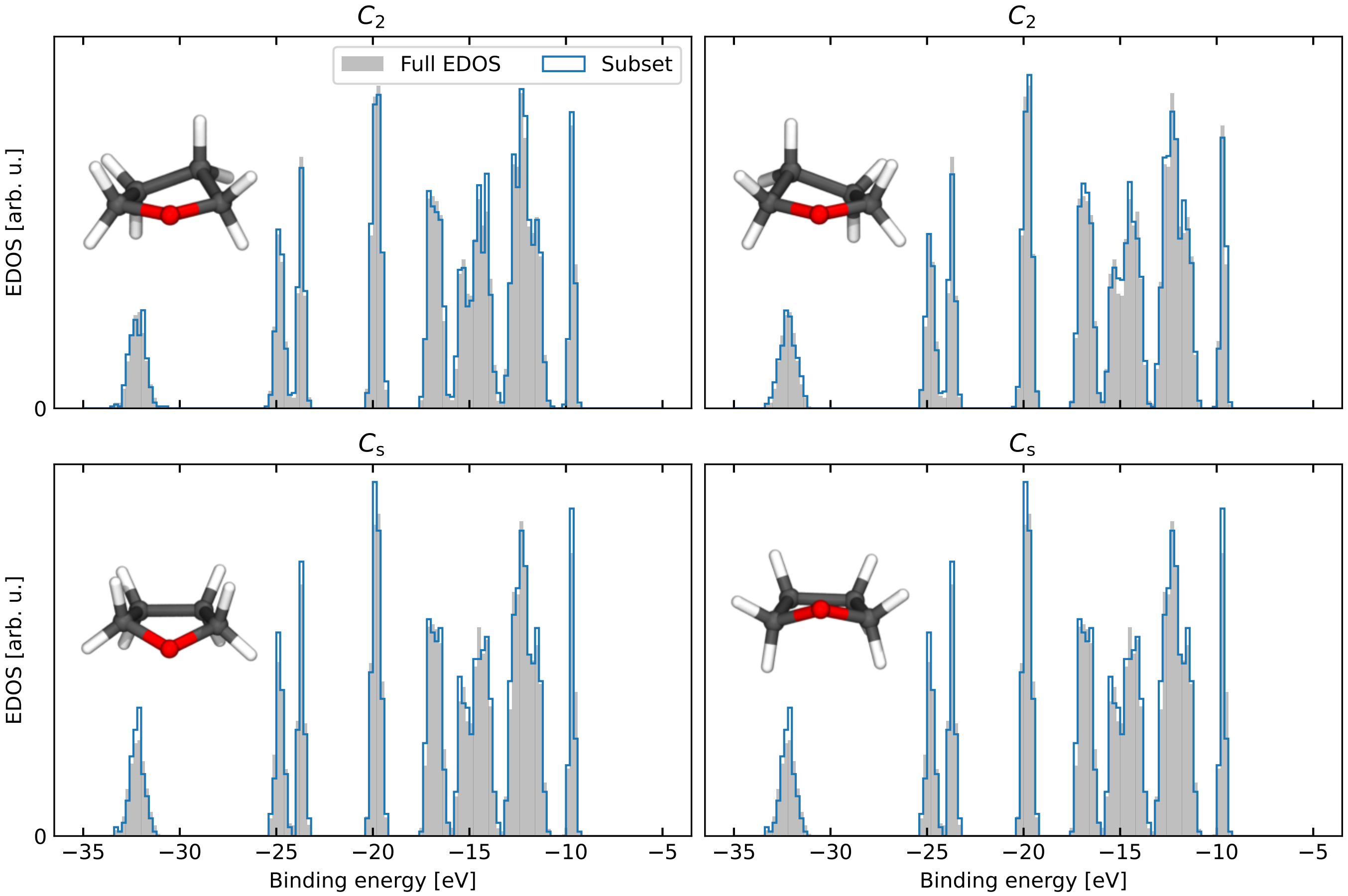


Figure S 2. Conformer-resolved EDOS. Each panel contains a snapshot of the representative cluster centroid, the EDOS calculated over the subset of configurations that belong to the same cluster as the centroid in blue and, as a reference, the total EDOS calculated over the whole set of configurations in gray.

on both instruments. We considered (1) the signal intensity of the HOMO of naphthalene using a fit compared to the background count rate, which determines the weakest signals that can be measured and (2) the noise using the interval to higher kinetic energies of naphthalene HOMO. We find that the naphthalene signal has a higher contrast to the background baseline in the helium lamp data and is recorded with comparable S/N to the synchrotron data. We can understand the contrast to baseline by noting that the monochromatized helium lamp radiation does not contain any higher harmonics which are present with the synchrotron/monochromator combination. A higher harmonic of the selected monochromator energy, while very small in absolute intensity, still cause undesired core-level excitations/ionizations. These contribute to a background count rate in the low electron binding energy region, which should be free of any electrons (except for the weakly bound electrons from the solutes we are interested in). This loss in contrast significantly impacts our sensitivity to detect electrons of interest.

We further noticed that we had a much higher sweep-to-sweep stability during the data acquisition at FHI compared to BESSY. We attribute this greatly to less charging of the THF jets upon illumination of the ionizing radiation no matter if the solutions of salts with $TBA^+PF_6^-$ or solutions of aromatic anions and alkali metal cations were used. Three characteristic differences result in a much more homogeneous illumination of the jet: (1) the Helium lamp emits light continuously while the synchrotron is a pulsed light source with pulse durations of several pico-seconds and repetition rates of MHz. (2) Using the 300 $\mu$m quartz capillary the Helium-plasma lamp has a specified photon flux of $\sim 1.8 \cdot 10^{11}$ photons/s and $\sim 1.0 \cdot 10^{10}$ for the He-I $\alpha$ (21.218 eV) and He-II $\alpha$ (40.814 eV) lines, respectively, compared to the synchrotron which has about 3-4 orders of magnitude higher photon flux. (3) The focal spot size of the Helium lamp is about 150 µm in diameter while the synchrotron focus is 60x80 µm $x$ µm. Even reducing the beamline slit sizes only resulted in a reduction but not a loss of the broadening due to charging.

A more significant advantage of the lab-based instrument is the availability of greater time to optimize experimental parameters in the flow jet. Particularly for solutions with dissolved alkali metals, this allowed us to increase the run time from $\sim$45 minutes to $\sim$150 minutes.

| | HOMO gas | HOMO liq | $\Delta G_{\mathbf{DFT}}$ | $\Delta G_{\mathbf{DFT}}$ range | $\Delta G_{\mathbf{BH}}$ |
|---|---|---|---|---|---|
| THF | -9.71 | -8.45 | 1.34 | 0.97 - 1.34 | 1.36 |
| Np | -8.19 | -7.28 | 0.91 | 0.90 - 0.95 | 0.94 |
| $Np^-$ | 0.007 | -2.48 | 2.47 | 2.39 - 2.64 | 2.66 |
| Bp | -9.04 | -8.30 | 0.74 | 0.74 - 0.86 | 0.78 |
| $Bp^-$ | -0.97 | -3.34 | 2.28 | 2.09 - 2.44 | 2.27 |

Table S 2. Solvent-induced shifts of the HOMO peak position (in eV) in the photo-electron spectrum, as determined by electronic structure calculations for each studied system, compared with the corresponding solvent shifts estimated using the classical Born-Haber cycle (last column).

This allowed to increase the effective acquisition time by a factor of 4 (from 30 to 120 minutes). A factor of 2 in S/N is gained by longer runtimes, as is evident in Figure 3 .

### C. Comparison of different cationic species in solutions of $Np^-$ in THF

In principle, all alkali metals are sufficiently strong reducing agents to form $Np^-$ in THF and we might expect similar PE spectra yielded from solutions formed from different alkali metals. However, it is known that the different alkalis give different ion pairing arrangements,[1] which might be expected to alter the $Np^-$ orbital energies in the weakly screening environment of THF. For example, lithium leads to solvent-separated pairs whereas sodium and potassium favors contact ion pairs. But it has also been observed that lithium is a strong enough reducing agent to reduce Np to a dianion, $Np^{2-}$.[6] On the topic of contact ion pairing, the literature also notes that there can be differences depending on the effective size of the excess charge in the anionic species: the larger cations contact pair with $Np^-$ while for the $Bp^-$ it is exactly the opposite: the small lithium cation is more likely to form contact ion-pairs.[7]

Spectra of $Np^-$ were recorded from various sample preparations while collecting data at the $SOL^3PES$ apparatus at BESSY. Figure S4 shows spectra where $Np^-$ is prepared from sodium, potassium and sodium/potassium alloy. Solutions of anions at ca. 0.3 M were possible but with some alkali metal still suspended in the solution. PE peaks seen in Figure S4 panel a) are most clearly observed in potassium solutions, with the PE spectra where Na and NaK alloy were used for reduction showing practically the same BE position for the two lowest spectral features.

### D. Estimation of the solvation free energy: PCM calculations and Born-Haber cycle

When probing the solute X, e.g., X being Np, via photoionization in a given solvent, the solute changes its oxidation number. In the case of an initial neutral state, X changes and becomes a cationic state, namely: $X^{\mathbf{Z=0}} \to X^{\mathbf{Z=+1}}$. In the case of an initial anionic state, X changes and becomes a final neutral state, namely: $X^{\mathbf{Z=-1}} \to X^{\mathbf{Z=0}}$. The solvation free energy $\Delta G$ can be estimated for both cases using the Born-Haber cycle.[8] The Born-model assumes the molecular species to be spherical with radius $R$ and an oxidation state $Z$ times the elementary charge $e$. The solvation free energy is thus given by the difference in energy to charge the sphere in a polarizable continuum $\varepsilon_r$ of the solvent versus the vacuum $\varepsilon_r = 1$. This leads to two distinct cases:[5]

(1) for an initial neutral species the final state is cationic: thus $\Delta G$ only needs to describe the electronic part of the solvent relaxation when stabilizing the nascent cationic solute. This is accounted for by replacing $\varepsilon_r$, by its high-frequency limit, which is equal to the square of the refractive index $n^2$.[5]

(2) for an initial anionic state the final state is neutral: here, $\Delta G$ is predominantly defined by the solvation of the anionic solute while the contribution from the final neutral is expected to be negligible. Thus the solvent polarizable continuum is given by its static dielectric constant which in THF exhibits a value of $\varepsilon = 7.42$.[9] Thus, the different initial oxidation states of the solute species interact with different parts of the dielectric constant, hence the difference in the solvation shift for neutral and anionic species.

The Born model is based on the assumption that the mechanical structure of charged entity in the polarizable continuum is spherical while the molecular structures of Np and Bp deviate quite significantly from a sphere. To account for that, we approximate average radii for Np and Bp by taking the mean value <R> between the maximum planar elongation (7.5 Å and 9.0 Å) and the minimum width $\approx$ 1 Å. Thus, the average radii are <R> $\sim$2.35 Å within the boundaries of [1 – 3.75 Å] for Np and <R> $\sim$2.75 Å within [1 – 4.5 Å] for Bp. This leads to a gas-liquid solvation shifts ranging from $\Delta G^{\mathrm{Np}^-}$ $\sim$2.66 eV [6.25 eV – 1.67 eV] and $\Delta G^{\mathrm{Bp}^-}$ $\sim$2.27 eV [6.25 eV – 1.39 eV]. We find that the average value agrees very well with the values found in the experiment as well as the theoretical DFT calculations. The $\Delta G$ associated with the more extreme values of the radii – the shorter as well as the longer limit – differ quite significantly which might well lie in the fact that these values reflect the deviation from the spherical entity the most.

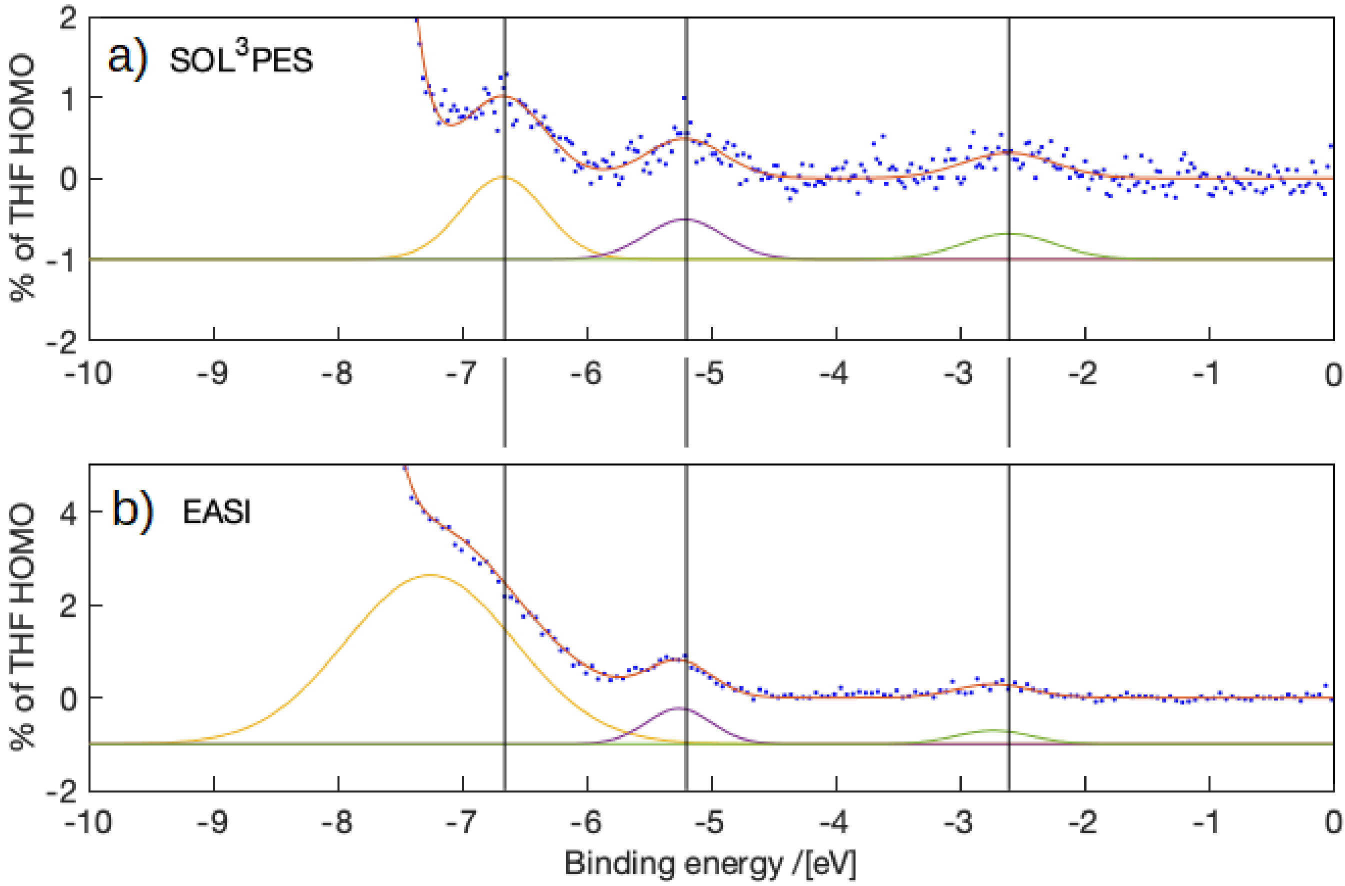


Figure S 3. Panel a): the liquid jet PE spectra for 0.4 M $Np^-$ in biased THF microjets, recorded on SOL$^3$PES at BESSY II (123.47 eV) and, panel b) on EASI with He-II $\alpha$ radiation (40.814 eV) using Na/K and K reduction of naphthalene, respectively. The vertical lines show the agreement between the two datasets. For two spectral features at binding energies, a single peak is consistently recovered near -2.7 and -5.5 eV. For the feature at higher binding energy before the spectrum becomes dominated by THF liquid HOMO spectral feature, the fit to the synchrotron dataset reveals a peak at -6.9 eV, whereas a larger and broader peak is apparent from the fit in the Helium lamp dataset centered at -7.4 eV. The overall fit of the three Gaussian fits is plotted in red through the experimental data (symbols) while the individual Gaussians are shown below with colors matching the color notation of the main paper

### E. Estimating VDE and reduction potential shifts as a function of the solvent's dielectric using the Born model

In the following the Born model is used to estimate the VDE shifts induced by the change of the solvent's dielectric. Based on our finding that the dominant contribution to the free energy of solvation is given by the change of the dielectric continuum we assume that the PE peak shape remains and the overall density of states shift depending on the changes of the dielectric constant $\varepsilon_r$. Thus the eBE of the peak centre of the HOMO (or SOMO) determines the VDE while the onset defines the ADE of a species. Choosing miscible solvents enables the generation of binary or ternary mixtures at different ratios to tailor $\varepsilon_r$. In the SI of Burrows et. al.[10] the ternary solution for the optimized Birch reduction of Np contains: 320 mmol of THF ($\varepsilon_r^{\mathrm{THF}} = 7.4$), 78 mmol EDA ($\varepsilon_r = 15.5$), and 23.4 mmol tert-butanol ($\varepsilon_r^{\text{t-BuOH}} = 12.5$). Assuming the solution's dielectric reflects the ratio of the number ratio of the individual solvent molecules one finds a $\varepsilon_r^{\mathrm{THF/EDA/t\text{-}BuOH}} = 9.2$ for the ternary solution. Under the same condition, a solution of EDA and tert-butanol without THF holds $\varepsilon_r^{\mathrm{EDA/t\text{-}BuOH}} = 14.2$. Using the Born-Haber model we estimate the free solvation energy to be $\Delta G_{\mathrm{BH}}^{\mathrm{THF/EDA/t\text{-}BuOH}} = 2.73$ eV and $\Delta G_{\mathrm{BH}}^{\mathrm{EDA/t\text{-}BuOH}} = 2.86$ eV. Thus, we estimate a shift to lower VDE of 0.13 eV (3 kcal/mol) by immersion in THF.

### F. Estimating the reduction potentials and thermodynamic stability of $Np^-$ and $Bp^-$ in THF

We use the PE spectra of $Np^-$ and $Bp^-$ in THF to evaluate the ADEs by estimating the onset at the low binding energy side of the lowest eBE spectral feature following the procedures described in Reference 11. Panels A, B and C of Figure S5 depict the separately measured

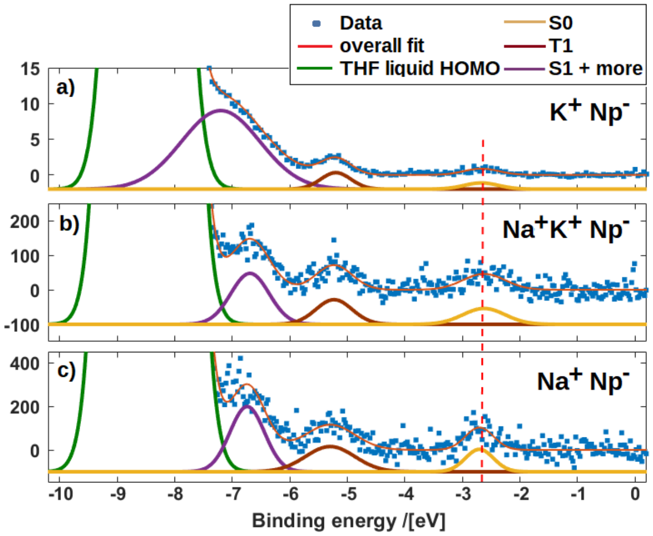


Figure S 4. Panels a) shows 0.3 mol/L $K^+$ $Np^-$ in THF recorded at FHI. Panels b), and c) show 0.3 mol/L $Na^+/K^+$ $Np^-$, and $Na^+$ $Np^-$ in THF recorded at BESSY, respectively.

PE spectra of the solutes of $Np^-$ and $Bp^-$ and ferrocene (Fc) in THF, respectively. The logarithmic PE signal intensities as a function of the eBE have been fit with a parabola since a parabola in the logarithmic plot represents a Gaussian function in linear plotting. The crossing point of the parabola with the noise level determines the onset value.[12]

We determine directly the $\mathrm{VIE}^{(\mathrm{Fc})}_{\mathrm{HOMO}}$ = 5.8 eV for Fc in THF. Moreover, we estimate, relative to vacuum, $^{(\mathrm{Np}^-)}\mathrm{ADE} = 2.21$ eV, $^{(\mathrm{Bp}^-)}\mathrm{ADE} = 2.80$ eV and $^{(\mathrm{Fc})}\mathrm{ADE}$ = 5.15 eV. The approach of self-consistently measuring Fc also by liquid-jet spectroscopy avoids needing to apply literature estimates of Fc with respect to the vacuum level, which enables us to estimate of the reduction potentials deirectly from the PE spectra, relative to a Ferrocene/THF standard as $E^0_{\mathrm{Np}^-} = -2.94$ eV and $E^0_{\mathrm{Bp}^-} = -2.35$ eV. These derived values agree well with those determined by cyclic voltammetry (CV), namely $^{\mathrm{CV}}E^0_{\mathrm{Np}^-} = -3.1$ eV and $^{\mathrm{CV}}E^0_{\mathrm{Bp}^-} = -2.3$ eV, respectively.[1] These results provide further confidence in our spectral assignments and the solution identity within the liquid microjet.

## G. Formation of different solute species in THF

The addition of alkali metal (for example: Li, Na, NaK, K, etc.) and aromatic compounds (e.g. benzene (Bz), Np, Bp, etc.) to the THF solvent allows in principle for the formation of various species. For example, the formation of $e^-_{\mathrm{solv}}$ in THF has been demonstrated by Seddon et al.[13] in flash photolysis experiments while experimental and theoretical studies by Schwartz[14,15] also investigated the dynamics of solvated electrons in liquid THF. Young et al.[16] extrapolated from cluster studies the VDE of the

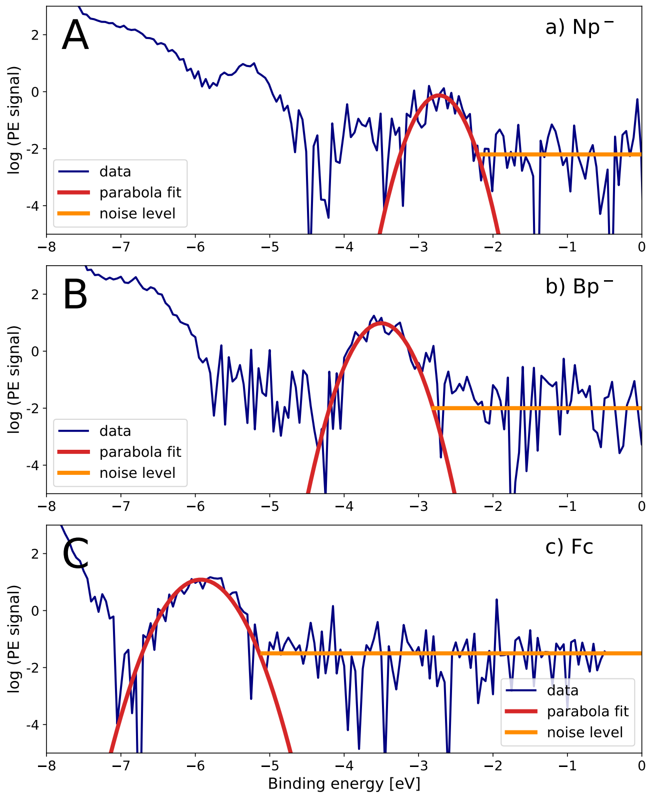


Figure S 5. Estimation of reduction potentials relative to the Ferrocene standard. Panels A, B, and C show the fits to the spectra of $Np^-$, $Bp^-$ and Fc in THF. Note the logarithmic scale to better perceive the peak onset.

$e^-_{\mathrm{solv}}$ in bulk THF to have a value of ~3.10 eV. All those investigations focused either on short time scales of less than a minute or used crown ethers to increase the solubility of the alkali metal while the extent of the solubility of alkali metals in THF is known to be rather limited. Down et al.[17] reported that the only alkali metal which has been found to cause a colour change from transparent to a pale blue solution is the sodium-potassium (NaK) alloy.

A competing process upon dissolution of M in THF is the disproportionation into alkali-metal anions ($M^-$) and cations ($M^+$). Also Seddon[13] et al. reported visible-NIR absorption spectra of alkalides formed in flash photolysis experiments which are compared to the absorption spectra of ion pairs of ($Na^+$, $e^-$) and ($K^+$, $e^-$) as well as of bare $e^-_{\mathrm{solv}}$ in THF. Notably, most experiments by Schwartz[18,19] and Ruhman[20] particularly focused on the formation the sodide ($Na^-$) anion in THF. For our present investigation on aromatic compounds in THF it is not *a priori* clear which species form: is it either $e^-_{\mathrm{solv}}$) or alkali metal ions ($M^-/M^+$) or aromatic radical anions? It could also happen that at thermodynamic equilibrium two or more species are present in solution, since parameters such as temperature or the time-scale can have a profound influence on the formation of certain species.

In order to determine which species form at what con-

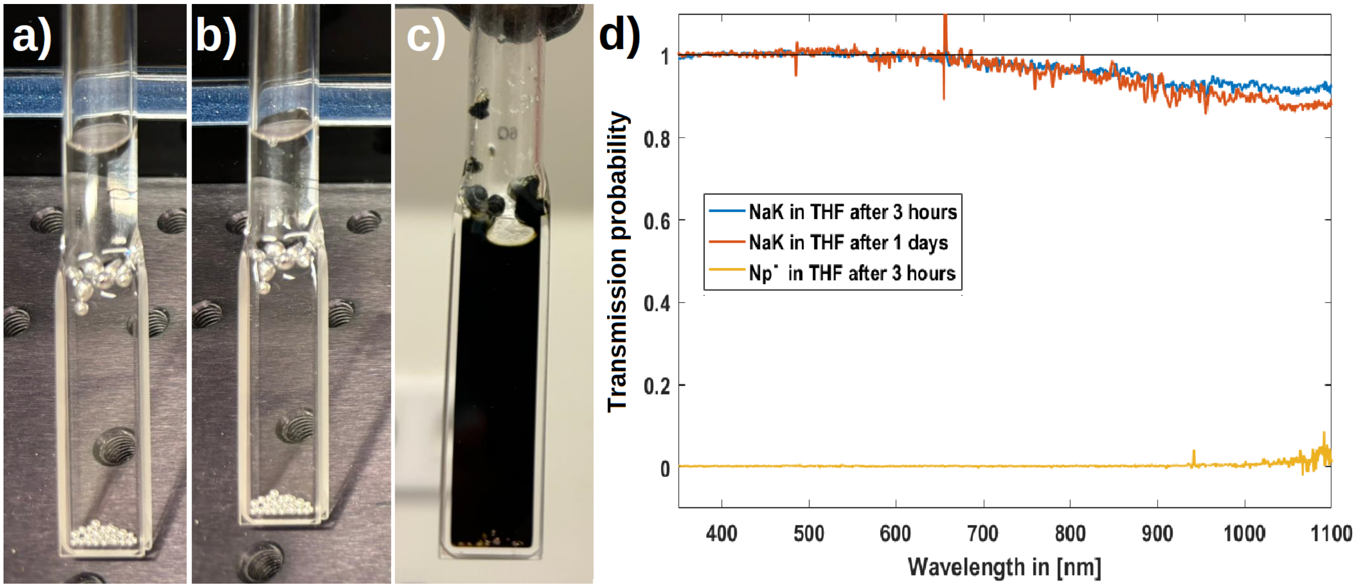


Figure S 6. Parts a), b) and c) show the NaK-THF and $Np^-$NaK-THF solution, respectively. Part d) depicts the corresponding absorption spectra.

centrations and on which time-scale of minutes to hours – relevant for the conduction of our experiment – we performed transmission absorption spectroscopy in the visible and the near IR between 350 – 1150 nm. The absorption of $Na^-$ and $K^-$ peak $\sim$ 800 nm and $\sim$ 900 nm, respectively, the absorption of the ion pairs of ($Na^+$, $e^-$) and ($K^+$, $e^-$) peaks $\sim$ 900 nm and $\sim$ 1100 nm, respectively, while the bare solvated $e^-$ absorbs around 2000 nm. Given the broad absorption bands of $\approx$ 1.5 eV of the investigated species, the absorption in the red and NIR range between 800 – 1200 nm should be observed if any of those species is formed.

Thus, we perfomed transmission absorption measurements of the THF-NaK solution in the visible-NIR region using a commercial white light source (AvaLight DHS) and a spectrometer (AvaSpec ULS2048xl-evo). For the sample solution making we used the same protocols as for the liquid jet experiments: for the solution making we used dried THF from a destillation unit and NaK drops which were contained and sealed in a 2 mm thick cuvette under an argon headspace. Figure 6 shows in the left parts the cuvette with the THF-NaK solution: a) 3 hours after making, b) 1 day after making and c) 3 hours after addition of Np to the 1 day old THF-NaK solution shown in part b). The right part d) shows the corresponding absorption spectra of the solution normalized by the spectral intensity distribution of the lamp (i.e. without the cuvette).

The absorption spectra of the THF-NaK solution (red and blue curves correspond to the solutions shown in parts a) and b), respectively) show a slight $\sim$10% absorption in the red and NIR range of the spectrum indicating the presence of $e^-_{solv}$ and/or $M^-$ species and/or ion pairs of ($Na^+$, $e^-$) and ($K^+$, $e^-$). The yellow curve shows the absorption spectrum of the $\sim$0.1 mol/L $Np^-$ solution shown in part c). In the following we will estimate the order of magnitude of the concentration of the various species using the Beer-lambert law: according to reference 13 the extinction coefficients of the $e^-_{solv}$, $Na^-$, $K^-$, ($Na^+$, $e^-$) and ($K^+$, $e^-$) are $5\times10^4$, $8.2\times10^4$, $10\times10^4$, $2.4\times10^4$ and $2.9\times10^4$ L/mol/cm, respectively, which are all within a factor of 2 relative to the $e^-_{solv}$ one. Given the transmission proability of 0.9 and the 2 mm length of the cuvette an a concentration of $\sim$ 1 mmol/L is estimated for $e^-_{solv}$ in solution. Thus, the $Np^-$ concentration in THF is at least 2 orders of magnitude higher than the $e^-_{solv}$ and/or $M^-$ species. Since we measured solutions of THF with $\sim$ 0.3 mol/L $Np^-$ and $Bp^-$ the species of $e^-_{solv}$ or $M^-$ or the ion pairs would be well below the detection sensitivity.

### H. Fit of experimental spectrum using theoretical binding energies as input

Figure S 7 depicts a comparison of the measured spectra which are fit using the theoretical electron binding energies of Table 2 of the main text as input parameters for an optimization routine. Each spectral feature is accounted for by a Gaussian function while the individual theoretical binding energies are the input for the center/mean values of the Gaussian. The fit-routine uses the sum of these Gaussian as an overall fit-function and optimizes the amplitudes/intensities and widths of the individual Gaussians to fit the experimental data best. Both left and both right graphs depict the experimental data of $Np^-$ in THF measured at BESSY and FHI, respectively. The fits in the upper and lower panels have been performed allowing a 0% error and a 5% error on the theoretical eBEs. The fits allowing up to 5% error

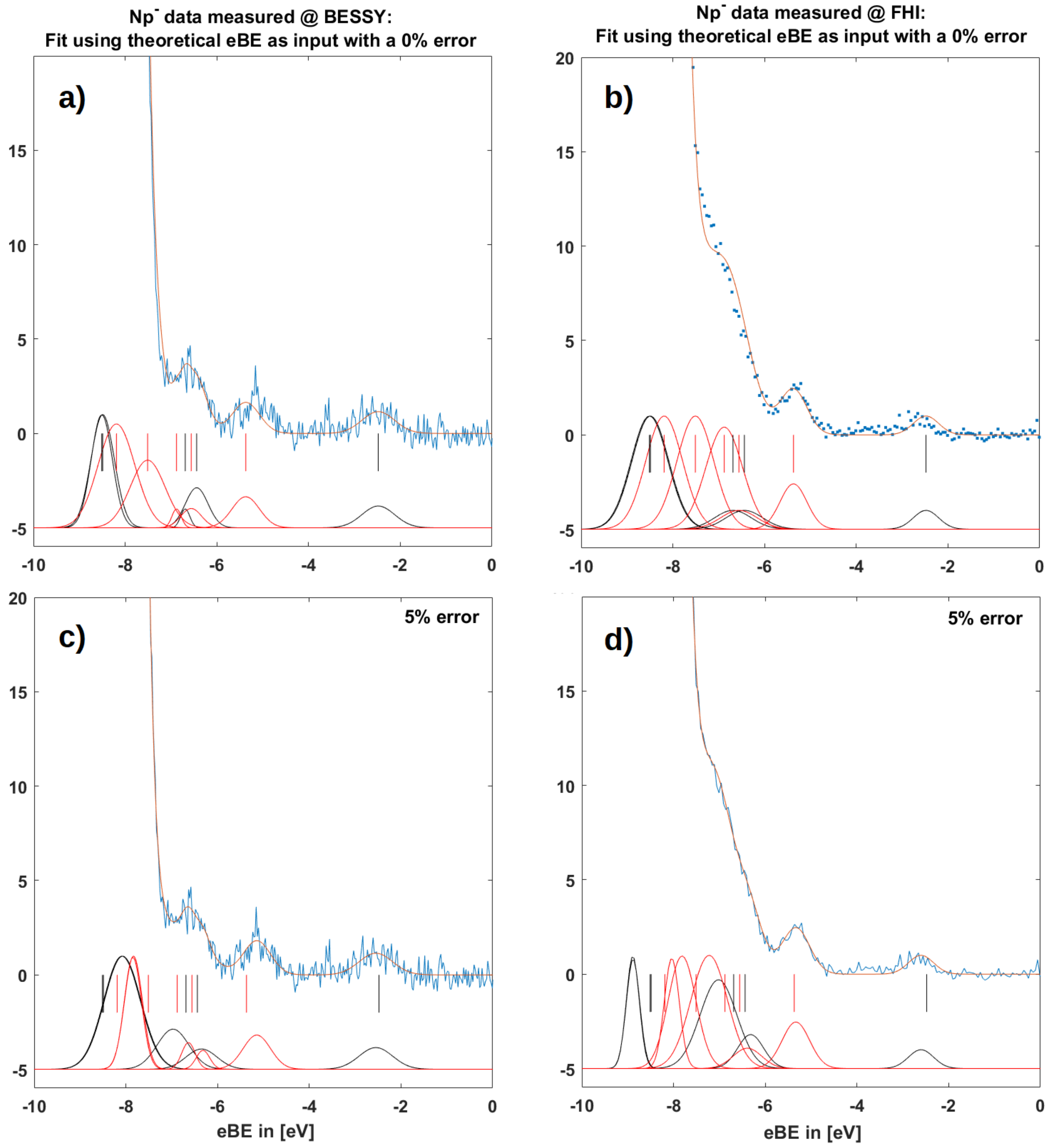


Figure S 7. Measured spectra are fit using the theoretical electron binding energies (The individual binding energies are listed in Table 2 of the main text) as input parameters for an optimization routine. Panels a) and b) depict the VB spectra of $Np^-$ in THF measured at BESSY and FHI, respectively. Panels c) and d) show the same measured data but allow a variance for the theoretical binding energies of up to 5%.

reproduce the data-sets almost perfectly leading to the conclusion: given the various sources of uncertainties and errors in both - the experiment and the calculation - we find agreement within an error of less than 5%.

## 4. THEORETICAL PROCEDURES

### A. Comments on the theoretical methods

Within our previous studies, we analyzed the aromatic radical anion of benzene using the density functional theory (DFT) and the perturbative Møller–Plesset (MP2) methods.[21,22] However, these single reference approaches were insufficient for a correct description of naphthalene radical anion and benzophenone radical anion. In particular, calculations revealed a multireference character of the investigated radical anions, as also manifested by high spin contamination of the MP2 results.

Given also the need to account for excited states for evaluating higher ionization energies and the fact that multireference-based approaches can in principle provide an accurate description thereof, we have opted to employ Complete Active Space Self-Consistent Field (CASSCF)[23] calculations using the ORCA 5.0.3 program package.[24,25] These multireference calculations revealed that for the naphthalene radical anion, the leading configuration accounted only for 84% of the ground state. For the ground state of the benzophenone radical anion, the multireference character was even more pronounced (77% contribution of the leading configuration).

Solvent effects were included in the form of a structureless medium within the integral equation formalism of the polarizable continuum model (IEF-PCM).[26–28] For a proper treatment of the solvation effect on fast photoionization processes, we employed the non-equilibrium PCM (NE-PCM) approach,[29,30] which accounts for a partial relaxation of the PCM cavity charges due to the high-frequency component of the dielectric constant $\varepsilon_\infty$ only.

### B. Spin density of $Np^-$ and $Bp^-$

Spin density is defined as the difference between the $\alpha$ electron density and the $\beta$ electron density. Electronic spin density is positive in areas where an electron is more likely to be found in the $\alpha$ spin state, and negative where an electron is more likely to be found in the $\beta$ spin state. In a radical molecule, the unpaired electron is most likely an $\alpha$ electron. This means that the spin density plot will show a predominant positive spin density, reflecting the excess of spin-up electrons compared to spin-down electrons. The small regions of negative spin density are often a consequence of the complexity of the radical's electronic structure (mostly delocalization) and can be also explained by the multiconfigurational character of the molecule arising from the mixing of electronic configurations that can lead to both positive and negative spin density regions in the molecule.

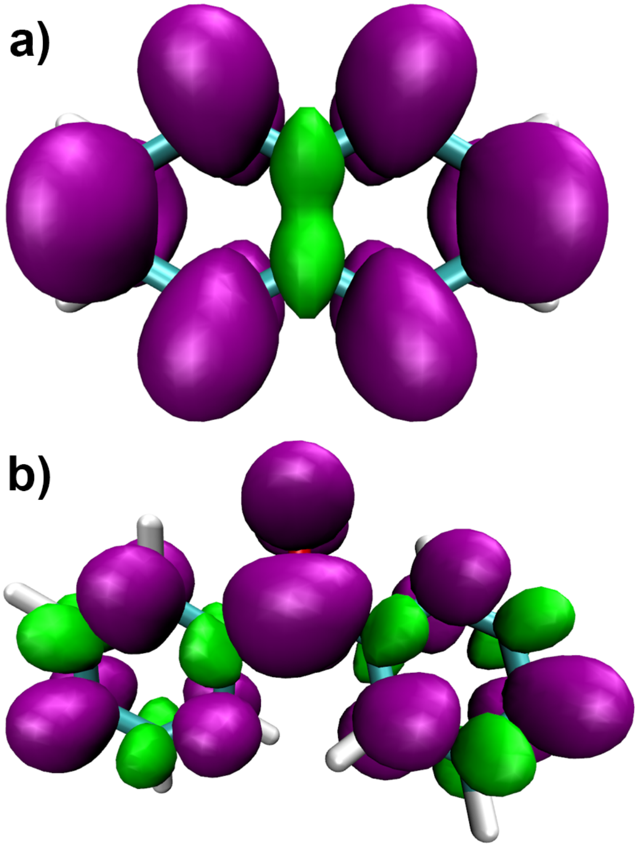


Figure S 8. Spin density and, thus the delocalization, of the excess electron on a) $Np^-$ and b) $Bp^-$ radical anions. Spin density is plotted with an isovalue of 0.001 $a_0^{-3}$ in purple for positive values and in green for negative.

### C. Does the spin density "leak" to the nearest solvent molecules?

No, it does not. Figure S9 shows the spin density of the studied aromatic radical anions with one explicit solvent molecule where no density can be identified on the solvent THF.

### D. Geometries of the radical anions and corresponding neutrals considered in this work

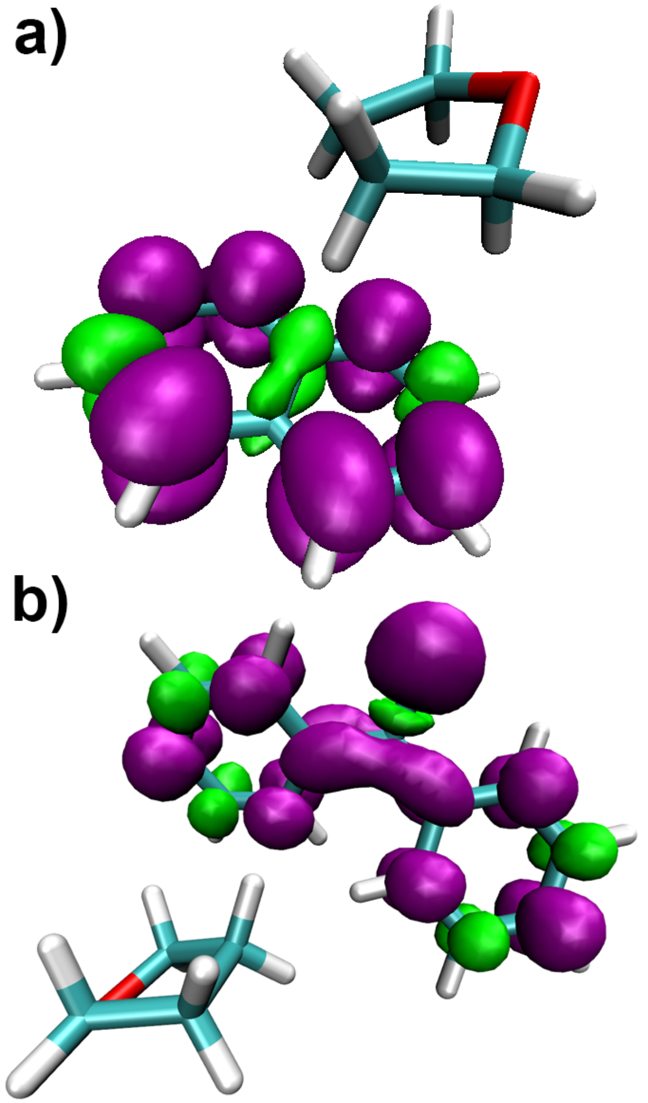


Figure S 9. Spin density of a) naphthalene radical anion and b) benzophenone radical anion with one explicit THF molecule. Spin density is plotted with an isovalue of 0.001 $a_0^{-3}$ in purple for positive values and in green for negative.

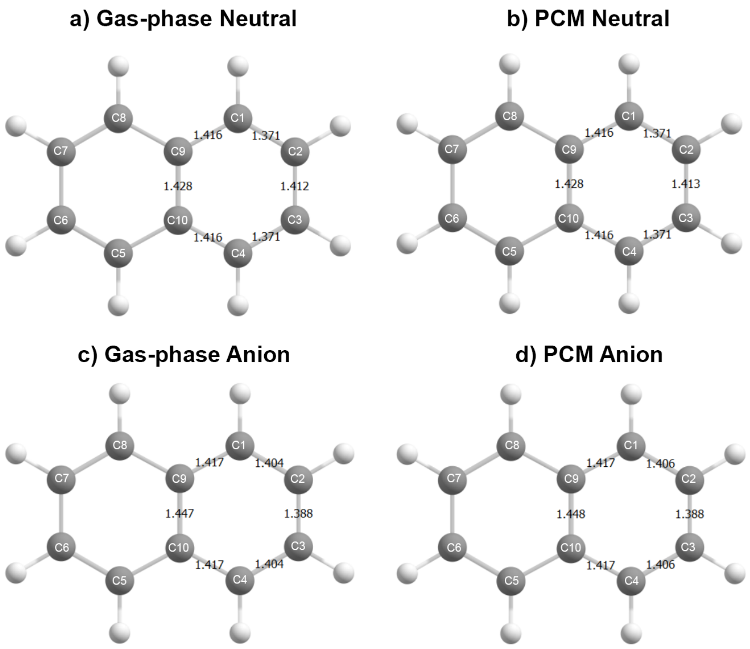


Figure S 10. Optimized geometries for $Np^-$ and Np, in the gas phase and within a polarizable continuum model. Note that the gas phase $Np^-$ is electronically unstable; the presented structure corresponds to a ”stabilization” calculation using a finite basis set.

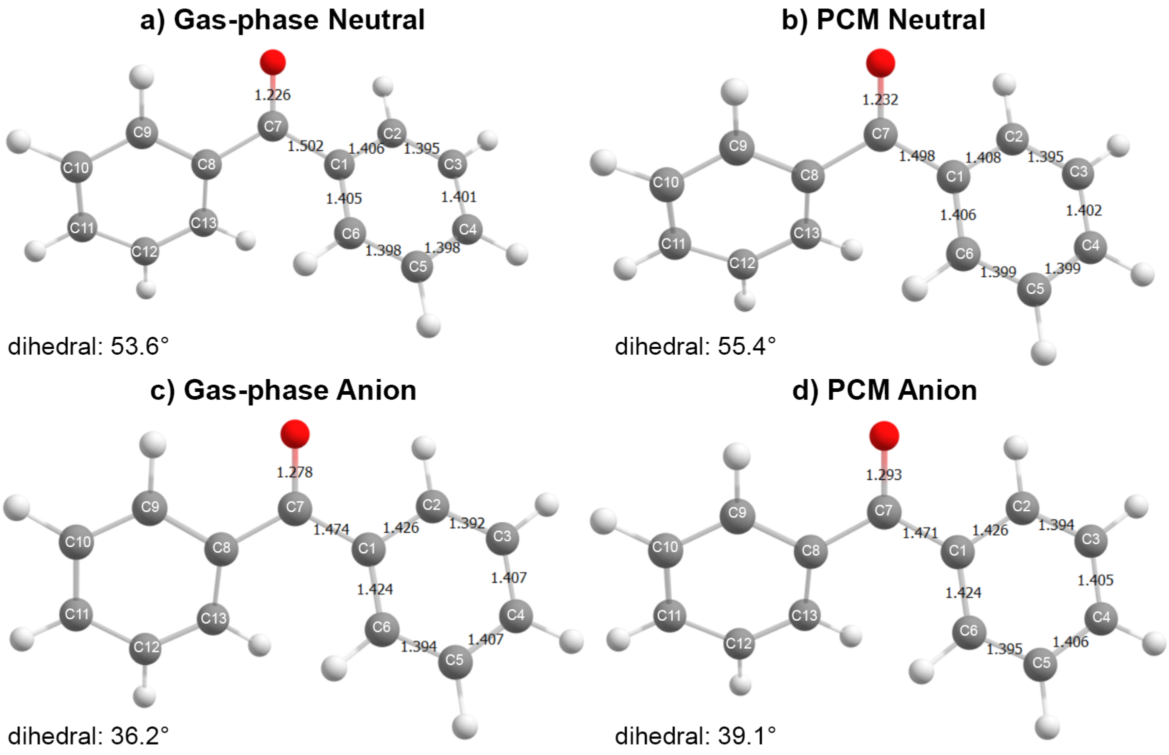


Figure S 11. Optimized geometries for $Bp^-$ and Bp, in the gas phase and within a polarizable continuum model.

### E. Molecular orbitals of naphthalene and benzophenone radical anions and leading determinants for each of the CAS calculations

| Energy above radical anion [eV] | Electronic determinants | |
|---|---|---|
| 2.48 (Neutral naphthalene ground state) | 78% | $(1b_{1u})^2$, $(1b_{2g})^2$, $(1b_{3g})^2$, $(1a_u)^2$, $(2b_{1u})^2$ |
| 5.37 | 66% | $(2b_{1u})^{-1}$ $(2b_{2g})^1$ |
| | 6% | $(1a_u)^{-1}$ $(3b_{1u})^1$ |
| 6.44 | 80% | $(2b_{1u})^{-1}$ $(2b_{2g})^1$ |
| 6.56 | 36% | $(1a_u)^{-1}$ $(2b_{2g})^1$ |
| | 34% | $(2b_{1u})^{-1}$ $(2b_{3g})^1$ |
| 6.69 | 36% | $(1a_u)^{-1}$ $(2b_{2g})^1$ |
| | 34% | $(2b_{1u})^{-1}$ $(2b_{3g})^1$ |
| 6.88 | 41% | $(1b_{3g})^{-1}$ $(2b_{2g})^1$ |
| | 33% | $(2b_{1u})^{-1}$ $(3b_{1u})^1$ |
| 7.51 | 64% | $(1a_u)^{-1}$ $(2b_{3g})^1$ |
| | 6% | $(2b_{1u})^{-1}$ $(2b_{2g})^1$ |
| 8.19 | 29% | $(1b_{2g})^{-1}$ $(2b_{2g})^1$ |
| | 17% | $(2b_{1u})^{-1}$ $(2a_u)^1$ |
| | 14% | $(1b_{3g})^{-1}$ $(2b_{3g})^1$ |
| | 12% | $(1a_u)^{-1}$ $(3b_{1u})^1$ |
| 8.49 | 21% | $(2b_{1u})^{-2}$ $(2b_{2g})^2$ |
| | 17% | $(1b_{3g})^{-1}$ $(2b_{3g})^1$ |
| | 14% | $(1a_u)^{-1}$ $(3b_{1u})^1$ |
| | 8% | $(1b_{2g})^{-1}$ $(2b_{2g})^1$ |
| 8.51 | 30% | $(1b_{3g})^{-1}$ $(2b_{2g})^1$ |
| | 19% | $(2b_{1u})^{-1}$ $(3b_{1u})^1$ |
| | 11% | $(1a_u)^{-1}$ $(b2_{1u})^{-1}$ $(2b_{2g})^2$ |
| | 8% | $(2b_{1u})^{-2}$ $(2b_{2g})^1$ $(2b_{3g})^1$ |

Table S 3. Dominant electronic determinants of the photoionization states from CAS-SCF calculation of excitation energies of neutral naphthalene. Only determinants with more than 5% contribution were included in the table. Those determinants accessible by one electron removal from the radical anion are shown in green.

| Energy above radical anion [eV] | Electronic determinants | |
|---|---|---|
| 3.34 (Neutral benzophenone ground state) | 72% | $(1b)^2$, $(1a)^2$, $(2b)^2$, $(3b)^2$, $(2a)^2$ $(4b)^2$, $(3a)^2$ |
| 6.86 | 45% | $(3a)^{-1}$ $(5b)^1$ |
| | 15% | $(3b)^{-1}$ $(4a)^1$ |
| 7.43 | 32% | $(3b)^{-1}$ $(5b)^1$ |
| | 18% | $(3a)^{-1}$ $(4a)^1$ |
| | 8% | $(4b)^{-1}$ $(6b)^1$ |
| | 8% | $(2a)^{-1}$ $(5a)^1$ |
| 7.64 | 26% | $(4b)^{-1}$ $(5b)^1$ |
| | 16% | $(2a)^{-1}$ $(4a)^1$ |
| | 8% | $(3b)^{-1}$ $(6b)^1$ |
| | 6% | $(3a)^{-1}$ $(5a)^1$ |
| 7.66 | 30% | $(2a)^{-1}$ $(5b)^1$ |
| | 16% | $(4b)^{-1}$ $(4a)^1$ |
| | 10% | $(3b)^{-1}$ $(5a)^1$ |
| | 10% | $(3a)^{-1}$ $(6b)^1$ |
| 7.89 | 22% | $(2a)^{-1}$ $(6b)^1$ |
| | 20% | $(4b)^{-1}$ $(5a)^1$ |
| | 7% | $(3a)^{-1}$ $(5b)^1$ |
| | 5% | $(4b)^{-1}$ $(5b)^1$ |
| | 5% | $(2b)^{-1}$ $(5b)^1$ |
| 7.98 | 26% | $(3b)^{-1}$ $(5b)^1$ |
| | 13% | $(2a)^{-1}$ $(4a)^1$ |
| | 10% | $(4b)^{-1}$ $(5a)^1$ |
| | 9% | $(3a)^{-1}$ $(6b)^1$ |
| 8.27 | 27% | $(2a)^{-1}$ $(5b)^1$ |
| | 13% | $(3b)^{-1}$ $(4a)^1$ |
| | 11% | $(4b)^{-1}$ $(6b)^1$ |
| | 9% | $(3a)^{-1}$ $(5a)^1$ |
| 8.74 | 37% | $(4b)^{-1}$ $(5b)^1$ |
| | 12% | $(4b)^{-1}$ $(3a)^{-1}(5b)^2$ |
| | 6% | $(3a)^{-2}$ $(5b)^1$ $(4a)^{-1}$ |
| 9.71 | 23% | $(3a)^{-2}$ $(5b)^2$ |
| | 10% | $(3a)^{-1}$ $(5b)^1$ |
| | 10% | $(4b)^{-1}$ $(3a)^{-1}$ $(5b)^1$ $(4a)^1$ |
| | 5% | $(2b)^{-1}$ $(5b)^1$ |

Table S 4. Dominant electronic determinants of the photoionization states from CAS-SCF calculation of excitation energies of neutral Bp. Only determinants with more than 5% contribution were included in the table. Those determinants accessible by one electron removal from the radical anion are shown in green.

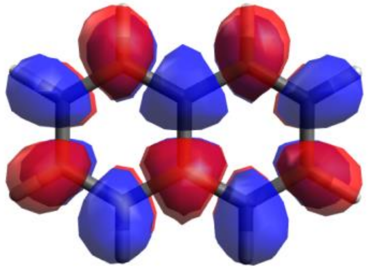

LUMO + 3
$3b_{3g}$

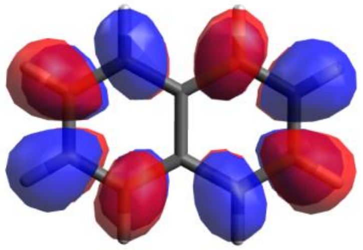

LUMO +2
$2a_u$

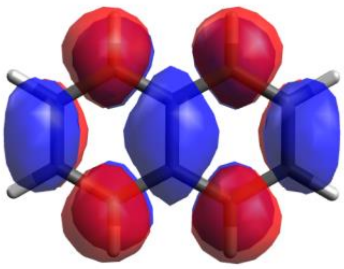

LUMO +1
$3b_{1u}$

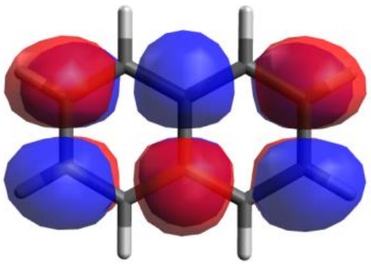

LUMO
$2b_{3g}$

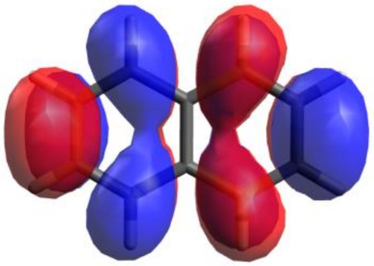

SOMO
$2b_{2g}$

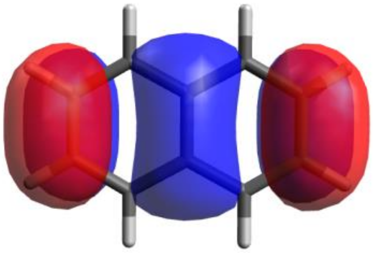

HOMO
$2b_{1u}$

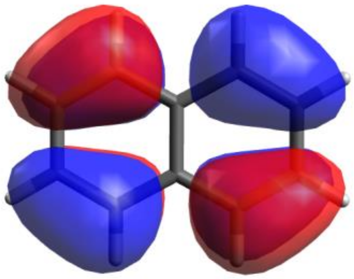

HOMO -1
$1a_u$

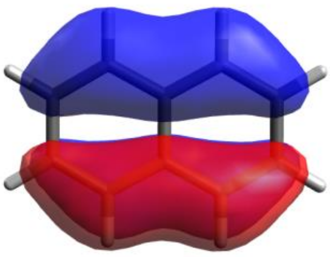

HOMO -2
$1b_{3g}$

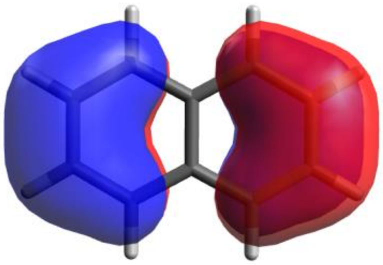

HOMO -3
$1b_{2g}$

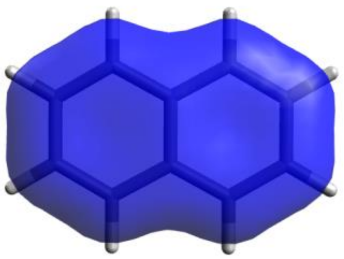

HOMO -4
$1b_{1u}$

Figure S 12. Molecular $\pi$ system of naphthalene radical anion. For simplicity reasons, the enumerating of the molecular orbitals started from the beginning of the active space ($\pi$ system).

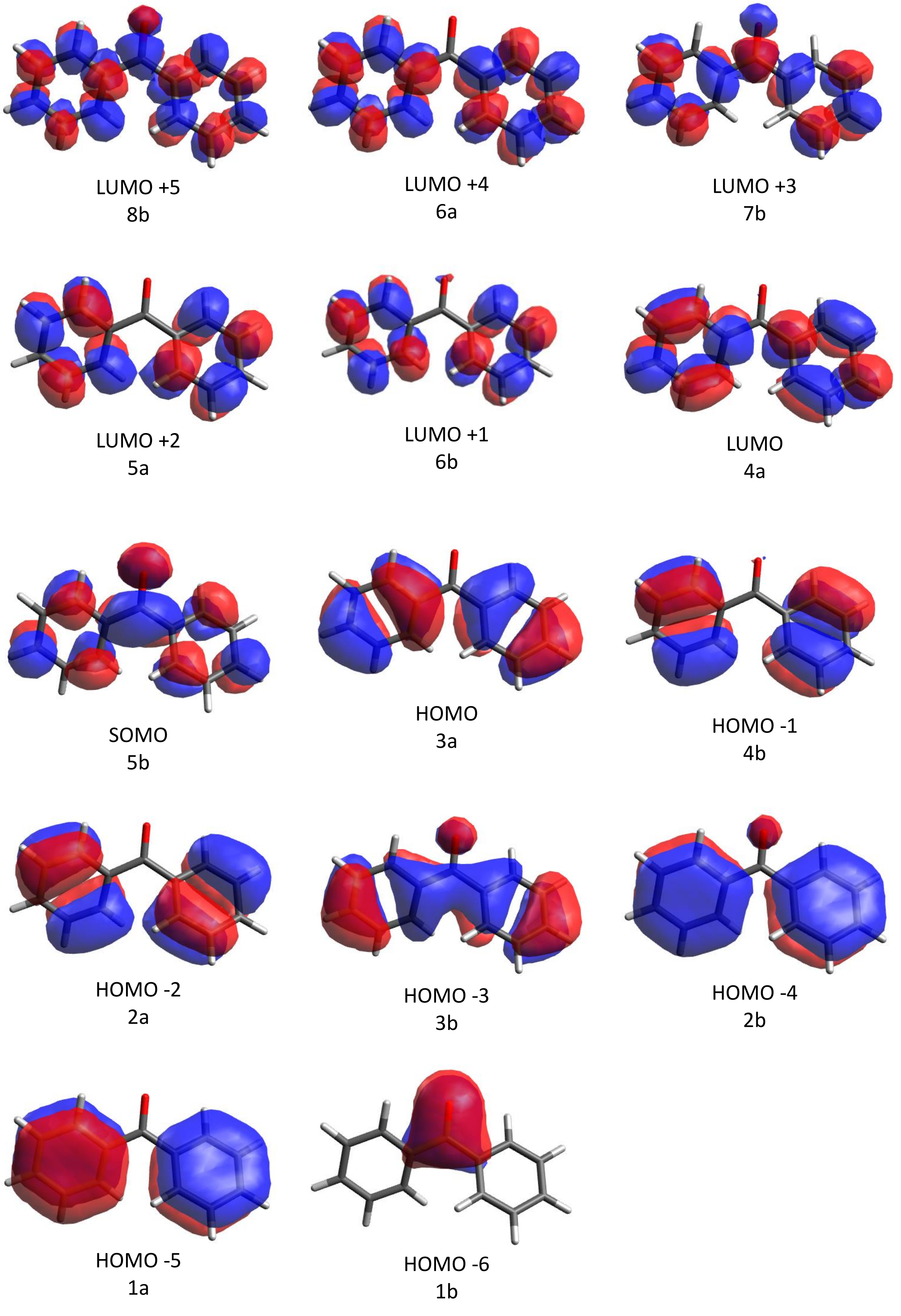


Figure S 13. Molecular $\pi$ system of Bp radical anion. For simplicity reasons, the enumerating of the molecular orbitals started from the beginning of the active space ($\pi$ system).

## REFERENCES


[1] N. G. Connelly and W. E. Geiger, "Chemical redox agents for organometallic chemistry," Chemical Reviews **96**, 877–910 (1996).

[2] J. Wilhelm, M. Del Ben, and J. Hutter, "Gw in the gaussian and plane waves scheme with application to linear acenes," Journal of Chemical Theory and Computation **12**, 3623–3635 (2016).

[3] F. Pedregosa, G. Varoquaux, A. Gramfort, V. Michel, B. Thirion, O. Grisel, M. Blondel, P. Prettenhofer, R. Weiss, V. Dubourg, *et al.*, "Scikit-learn: Machine learning in python," the Journal of machine Learning research **12**, 2825–2830 (2011).

[4] S. Thuermer, S. Malerz, F. Trinter, U. Hergenhahn, C. Lee, D. Neumark, G. Meijer, B. Winter, and I. Wilkinson, "Accurate vertical ionization energy and work function determinations of liquid water and aqueous solutions," Chemical Science **12**, 10558–10582 (2021).

[5] H. C. Schewe, K. Brezina, V. Kostal, P. E. Mason, T. Buttersack, D. M. Stemer, R. Seidel, W. Quevedo, F. Trinter, B. Winter, *et al.*, "Photoelectron spectroscopy of benzene in the liquid phase and dissolved in liquid ammonia," The Journal of Physical Chemistry B **126**, 229–238 (2021).

[6] J. Smid, "A stable dianion of naphthalene," Journal of the American Chemical Society **87**, 655–656 (1965).

[7] R. G. Pearson, "Absolute electronegativity and hardness: application to inorganic chemistry," Inorganic Chemistry **27**, 734–740 (1988).

[8] B. Winter and M. Faubel, "Photoemission from liquid aqueous solutions," Chemical Reviews **106**, 1176–1211 (2006).

[9] Z. Baird, P. Uusi-Kyyny, J. Pokki, and et al., "Vapor pressures, densities, and pc-saft parameters for 11 bio-compounds," Int. J. Thermophysics **40** (2019), 10.1007/s10765-019-2570-9.

[10] J. Burrows, S. Kamo, and K. Koide, "Scalable Birch reduction with lithium and ethylenediamine in tetrahydrofuran," Science **374**, 741–746 (2021).

[11] X.-B. Wang and L.-S. Wang, "Probing the electronic structure of redox species and direct determination of intrinsic reorganization energies of electron transfer reactions," The Journal of Chemical Physics **112**, 6959–6962 (2000).

[12] A. Roy, R. Seidel, G. Kumar, and S. E. Bradforth, "Exploring redox properties of aromatic amino acids in water: Contrasting single photon vs resonant multiphoton ionization in aqueous solutions," The Journal of Physical Chemistry B **122**, 3723–3733 (2018).

[13] W. A. Seddon, J. W. Fletcher, F. C. Sopchyshyn, and E. B. Selkirk, "Flash photolysis of alkali metal anions in tetrahydrofuran and dimethoxyethane," Canadian Journal of Chemistry **57**, 1792–1800 (1979).

[14] M. J. Bedard-Hearn, R. E. Larsen, and B. J. Schwartz, "The role of solvent structure in the absorption spectrum of solvated electrons: Mixed quantum/classical simulations in tetrahydrofuran," The Journal of Chemical Physics **122**, 134506 (2005).

[15] I. B. Martini, E. R. Barthel, and B. J. Schwartz, "Optical control of electrons during electron transfer," Science **293**, 462–465 (2001).

[16] R. M. Young and D. M. Neumark, "Dynamics of solvated electrons in clusters," Chemical Reviews **112**, 5553–5577 (2012).

[17] J. L. Down, J. Lewis, B. Moore, and G. Wilkinson, "761. the solubility of alkali metals in ethers," J. Chem. Soc. , 3767–3773 (1959).

[18] A. E. Bragg, M. C. Cavanagh, and B. J. Schwartz, "Linear response breakdown in solvation dynamics induced by atomic electron-transfer reactions," Science **321**, 1817–1822 (2008).

[19] E. R. Barthel, I. B. Martini, and B. J. Schwartz, "Direct observation of charge-transfer-to-solvent (CTTS) reactions: Ultrafast dynamics of the photoexcited alkali metal anion sodide," The Journal of Chemical Physics **112**, 9433–9444 (2000).

[20] O. Shoshana, J. L. Pérez Lustres, N. P. Ernsting, and S. Ruhman, "Mapping ctts dynamics of na- in tetrahydrofurane with ultrafast multichannel pump-probe spectroscopy," Phys. Chem. Chem. Phys. **8**, 2599–2609 (2006).

[21] K. Brezina, V. Kostal, P. Jungwirth, and O. Marsalek, "Electronic structure of the solvated benzene radical anion," The Journal of Chemical Physics **156**, 014501 (2022).

[22] T. Nemirovich, V. Kostal, J. Copko, H. C. Schewe, S. Bohacova, T. Martinek, T. Slanina, and P. Jungwirth, "Bridging electrochemistry and photoelectron spectroscopy in the context of birch reduction: Detachment energies and redox potentials of electron, dielectron, and benzene radical anion in liquid ammonia," Journal of the American Chemical Society **144**, 22093–22100 (2022).

[23] B. O. Roos, "Multiconfigurational quantum chemistry," in *Theory and Applications of Computational Chemistry* (Elsevier, 2005) Chap. 25, pp. 725–764.

[24] F. Neese, "The orca program system," Wiley Interdisciplinary Reviews: Computational Molecular Science **2**, 73–78 (2012).

[25] F. Neese, "Software update: The orca program system—version 5.0," Wiley Interdisciplinary Reviews: Computational Molecular Science **12**, e1606 (2022).

[26] E. Cances, B. Mennucci, and J. Tomasi, "A new integral equation formalism for the polarizable continuum model: Theoretical background and applications to isotropic and anisotropic dielectrics," The Journal of chemical physics **107**, 3032–3041 (1997).

[27] J. Tomasi, B. Mennucci, and E. Cancès, "The ief version of the pcm solvation method: an overview of a new method addressed to study molecular solutes at the qm ab initio level," Journal of Molecular Structure: THEOCHEM **464**, 211–226 (1999).

[28] J. Tomasi, B. Mennucci, and R. Cammi, "Quantum mechanical continuum solvation models," Chemical reviews **105**, 2999–3094 (2005).

[29] M. Cossi and V. Barone, "Solvent effect on vertical electronic transitions by the polarizable continuum model," The Journal of Chemical Physics **112**, 2427–2435 (2000).

[30] M. Cossi and V. Barone, "Separation between fast and slow polarizations in continuum solvation models," The Journal of Physical Chemistry A **104**, 10614–10622 (2000).
